# Intelligent MPPT and coordinated control for voltage stability in brushless DFIG wind turbines

**DOI:** 10.1038/s41598-025-08676-x

**Published:** 2025-07-02

**Authors:** Prashanth Rajanala, Malligunta Kiran Kumar, Ambati Giriprasad, Joon-Ho Choi, K. V. Govardhan Rao, V. Sri Sravan, Ch. Rami Reddy

**Affiliations:** 1 Department of Electrical and Electronics Engineering, Koneru Lakshmaiah Education Foundation, Guntur, India; 2 Department of Electrical and Electronics Engineering, VNR Vignana Jyothi Institute of Engineering and Technology, Hyderabad, 500090 India; 3 Electrical Engineering, Chonnam National University, Gwangju, South Korea; 4 Electrical and Electronics Engineering, St. Martin’s Engineering College, Secunderabad, Telangana India; 5 Department of Computer Science and Engineering, Koneru Lakshmaiah Education Foundation, Guntur, India; 6 Applied Science Research Center, Applied Science Private University, Amman, 11937 Jordan; 7 Department of Electrical and Electronics Engineering, Joginpally B R Engineering College, Hyderabad, 500075 India

**Keywords:** Wind energy conversion system (WECS), Doubly fed induction generator (DFIG), Grid side control (GSC), Rotor side control (RSC), Chaotic salp swarm optimisation (CSSO-ANFIS) - based MPPT, Energy science and technology, Renewable energy

## Abstract

This research develops a novel control approach for improving voltage stability and maximizing power extraction in Brushless Doubly Fed Induction Generator (DFIG) based Wind Energy Conversion Systems (WECS). The developed approach incorporates a Chaotic Salp Swarm Optimization (CSSO) tuned Adaptive Neuro Fuzzy Inference System (ANFIS) for Maximum Power Point Tracking (MPPT) allowing rotor speed’s dynamic adjustments and torque to attain better wind power extraction. The control framework has coordinated control among the Rotor Side Converter (RSC) and Grid Side Converter (GSC), where the GSC provides the delivery of power to the grid and offers grid support features while the RSC manages torque of rotor side and DC link voltage. To support grid stability under changing conditions, reactive power balancing and voltage regulation are incorporated into the system. By utilizing a d-q reference frame based current control strategy, the harmonic distortion in the grid current is alleviated. Furthermore, the efficacy of developed controller is validated in MATLAB/Simulink tool demonstrating tracking efficiency of $\:99.86\:\%,$ with improved tracking speed (0.08s), reduced total harmonic distortion (THD < 2.85%), enhanced voltage stability revealing significant improvements in voltage stability, harmonic suppression and wind energy harvesting efficiency under both steady-state and dynamic operating conditions.

## Introduction

Growing emphasis on renewable energy generation is a result of the world’s rising energy demands as well as the severity of climate change. Wind energy has become as a major option that is expanding quickly in the globe for renewable energy^1–4^. Its rapid evolution has positioned it as a viable solution for clean energy production. Wind energy generation has recently garnered international attention as an environmentally beneficial power generation technology that harnesses wind energy to produce electricity. A number of causes, including the depletion of fossil fuels, growing public awareness of environmental deterioration, and developments in environmentally friendly technologies, have contributed to the tremendous growth of wind energy over the previous 20 years, as part of the renewable energy industry. However, the amount of energy produced is heavily influenced by meteorological conditions, particularly wind speed^5–10^.

Traditional WECSs typically use gearbox drive trains to power either a PMSG or a DFIG. However, gearbox failures can significantly increase maintenance costs and downtime, making them a critical issue for traditional WECSs. DFIGs have emerged as a preferred option for wind turbine uses because of their dependability and great efficiency^11–15^. Their ability to operate at variable speeds further enhances their efficiency in capturing energy from varying wind speeds^16– 17^. The dual feed induction generator is a well-liked substitute for variable-speed wind turbine configurations due to its durability and little wear on mechanical components. The advantage of both power of active and reactive control is offered by these generators. However, when the generator, the rotor and the grid are connected using back-to-back converters, there are restrictions on the power transfer capacity^18–25^. DFIGs have a partially rated converter providing power to the rotor winding addition to multi-stage engine. When wind speeds exceed the rated speed, pitch adjustment is used to maintain the rated power output. DFIGs may supply the rated active power for the majority of operating hours when 25 − 30% of the rated power is the converted power rate^25–29^. DFIGs connecting grid through direct links, where the rotor is connected to RSCs and GSCs via a DC-link, resembles the configuration of standard wound-type induction machines^26,27^^30^–^32^.

In WECSs, actual MPPT controllers are essential for preserving the maximum power point in any kind of environment. The improved Incremental conductance^33^, fuzzy logic controller^29^, artificial neural network^30^, perturb and observe^31^, and ANFIS-based are just a few of the MPPT controllers that have been developed^34^. Drawbacks include complexity in design and training, the need for large training data, significant computational resources, risk of overfitting and challenges in interpretability. To address the limitations of traditional methods, this study introduces an innovative^23,35–37^ AI-driven MPPT method known as the CSSO-ANFIS MPPT approach, aiming to optimize power extraction efficiency from wind turbines. By using this approach, the learning capacity with generalization is balanced leading to the minimization of overfitting risks. Moreover, the CSSO algorithm automates the ANFIS parameter optimization generating a simplified design process. Also, CSSO has the ability to fine-tune the system parameters and improve the performance of the model with smaller datasets thus preventing over-reliance on training data. By proving global optimization^38–42^, CSSO reduces computational overhead and converges quickly to optimal solutions and the interpretability of the control strategy is improved by the fuzzy inference system. Therefore, this research develops the CSSO-ANFIS MPPT approach to enhance power extraction efficacy from wind turbines.

**Table 1:** Comparison of advanced MPPT approaches for wind System.

S.no	Author/Year of Publication	Methodology Used	Merits	Demerits
1	^50^Mohammad Mahdi Rezaei et al., (2018)	Adaptive Backstepping Control Method For MPPT In DFIG-Based WECS.	Robustness, Sensor control and Adaptive control	Slow response time
2	Sobhy S. Dessouky et al., (2018)^51^	Perturb and Observe technique for MPPT	Cost-Effective, Efficient Power Extraction and Simulation-Based Validation	Sensitivity to Parameter Variations
3	Yuliang Sun et al., (2018)^52^	Coordination of Feedback Linearization Techniques (CFLS) for DC-oriented DFIG system to track the MPP.	Single-Loop Control, Decoupling Control and Superior Performance.	Limited adaptability to changing operating conditions.
4	Essam.H.Abdouet al., (2019)^53^	Adaptive Perturb and Observe (AD-PO) MPPT	Improved Performance, Adaptive Step Size and Validation	Difficulty in tuning parameters for different wind conditions.
5	S. Souedet al., (2019)^54^	Metaheuristic Optimization Techniques (MOTs): The ABC algorithm and GWO are two examples of MOTs algorithm to achieve MPPT for the WECS	Enhanced MPPT, Dynamic Performance Improvement and Simulation Validation	Potential for convergence to suboptimal solutions.
6	Arjun Kumar GB et al., (2020)^55^	Implementing Algorithms for the MPPT of wind and solar energy	Reduced Grid Dependence, Efficiency Improvement, Grid Stability and Environmental Benefits	Increased complexity in system integration.
8	Muhammad Zafran et al., (2020)^56^	Fast Dynamic Terminal Sliding Mode Control (Fdtsmc)-Based Maximum Power Point Tracking (Mppt)	Optimized Power Extraction, Performance Comparison, Global Robustness and Simulation Validation	Potential for instability under sudden load changes.
9	Jie Wang et al., (2022)^57^	Fixed-Time Observer, Optimal Torque Control, Adaptive Controller for Fixed-Time Non-singular Terminal Sliding Mode and Stability Analysis	Elimination of Wind Speed Sensors, Robustness and Efficiency	Higher computational resource requirements
10	Shivaji Ganpat Karad et al., (2022)^58^	A modified A FOPID controller-based incremental conductance (INC) For a wind turbine system based on a doubly fed induction generator (DFIG), a maximum power point tracking (MPPT) controller is recommended	Efficiency, Optimization and Adaptability	Challenges in maintaining stability under varying wind conditions
11	Boni Satya Varun Sai et al., (2022)^59^	Swarm Optimization of Particles (SSM-PSO): A Proposed Method for Tracking Maximum Power Points (MPPT) in a DFIG-based web-enabled communications system	Improved Dynamic Characteristics, Weather Insensitive and Faster Response	Increased convergence time.
12	Sara Kadi et al., (2023)^60^	DFIG-based wind power conversion systems with dependable nonlinear chattering-free third-order sliding mode control (TOSMC).	Chattering-Free Control, Improved Power Quality, Enhanced Response Time and Robustness	Complexity in implementation and tuning.
13	Jang-Hyun Park et al., (2023)^61^	Higher-Order Switching Differentiator (HOSD-based MPPT controller for a WECS with a DFIG	Reduced Need for System Information, Simplified Controller Design and Efficient MPPT and Reactive Power Regulation	Limited robustness to variations in wind speed.

Table 1 compares existing methods with their merits and demerits along the features enabled in this research work. The research work includes optimized MPPT control, enhanced system effectiveness, rotor speed and torque control, grid-side control loop, grid support functions, and harmonic mitigation, which aim to get around the drawbacks of the current approaches. Overall, this work contributes a novel CSSO-ANFIS for the maximum power point tracking in a based wind energy conversion system by addressing limitations like slow convergence and overfitting. The torque and rotor speed are adjusted for maximizing power extraction efficiency resulting in reduced energy losses. The d-q transformation is employed for generating reference currents for minimizing harmonic distortion in grid current. Thus the proposed work demonstrates a scalable and practical solution to improve the efficiency and reliability of wind energy systems.

The major contributions of this article are:

- Development of a CSSO-ANFIS-based MPPT that adaptively tunes control parameters to track the maximum power point efficiently, even under rapidly changing wind profiles.
- Implementation of a coordinated RSC and GSC control strategy that enables not only efficient power transfer but also provides grid support functions such as voltage regulation and harmonic mitigation.
- Demonstration of superior performance through MATLAB/Simulink simulations, including reduced tracking error, faster convergence (0.08s), low THD (< 2.85%), and improved power quality, highlighting the robustness of the proposed controller in dynamic conditions.

The paper’s structure is as follows: In Sect. 1, the Introduction part is given. In Sect. 2, the WECS concept is provided. Section 3 provides a detailed description of the grid-connected DFIG-based WECS system setup as well as the modelling of doubly-fed induction generators and wind turbines. Section 4 presents the result and discussion. In Sect. 5, the research is concluded.

## Proposed methodology

**Fig. 1:**
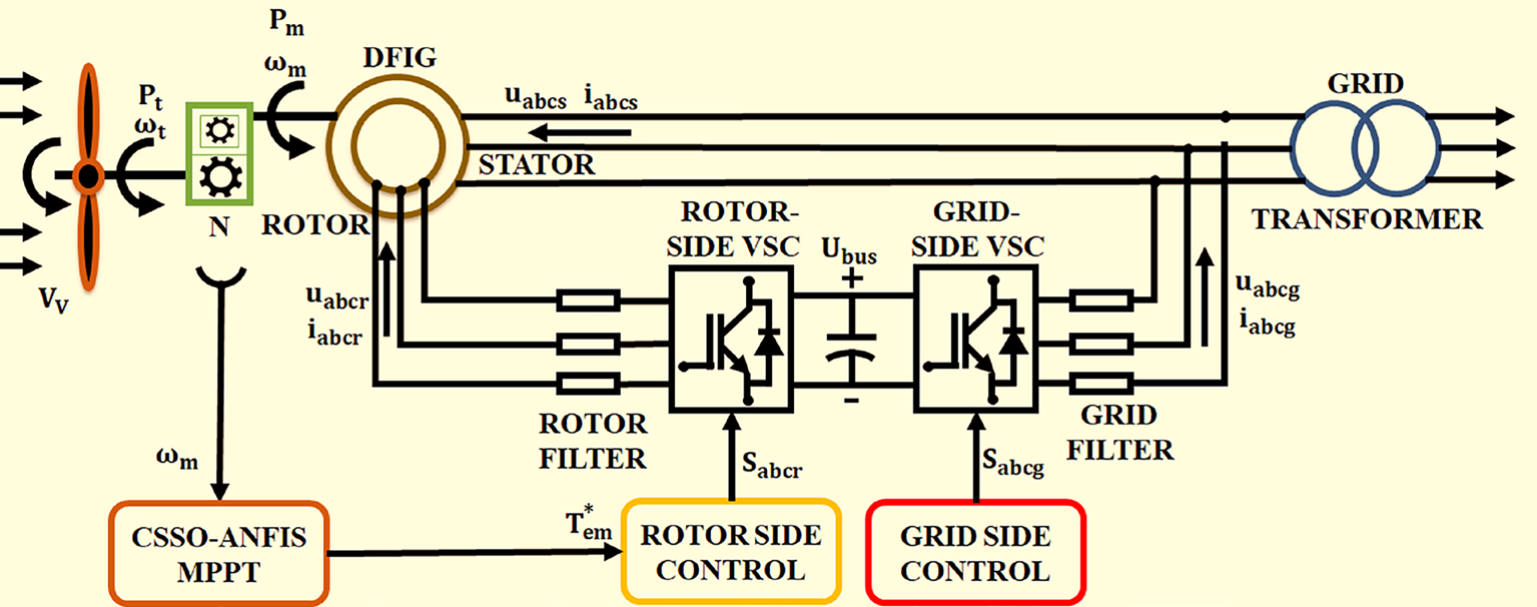
Description of the proposed Model.

The depicted system in the following Fig. 1 is a sophisticated setup for a WECS-enabling DFIG. The model initiates the wind turbine harnessing kinetic energy, translating it into mechanical motion that drives the DFIG. The generator has two key components: the rotor, which connects the grid via the RSC and GSC, and the stator, which is directly connected to the grid. In essence, RSC is tasked with managing the voltage at the DC link by adjusting the rotor’s electromagnetic fields, which inherently changes the electrical characteristics of the DFIG. Through this action, the RSC indirectly controls the power generated, aligning it with the optimal power point tracked by the CSSO-ANFIS MPPT system. In order to guarantee that the wind turbine functions at its most efficient position regardless of fluctuating wind conditions, the MPPT is a crucial part of wind energy systems. In the CSSO-ANFIS-based MPPT strategy, two advanced techniques are amalgamated to create a highly adaptive and efficient control system. The CSSO method draws inspiration from salps’ natural swarming behaviour, incorporating chaotic variables to avoid local minima and enhance the global search capability. This algorithm is adept at handling the nonlinear and unpredictable nature of wind profiles, swiftly converging to the optimal solution even in the presence of rapid wind speed variations. ANFIS learns the ideal power curve of the wind turbine over time and creates a fuzzy inference system that can predict the ideal operating points in various scenarios. The functioning of MPPT involves continuously monitoring the wind speed, the rotor’s angular velocity, and the generator’s power output^43–49^. The CSSO part of the algorithm explores possible solutions, while the ANFIS refines these solutions based on learned data to predict the precise generator of the pace at which the MPPT is reached. The output of MPPT denotes a reference value for the rotor’s angular velocity that is communicated to the RSC^50–56^. The RSC uses this reference to adjust the generator’s excitation, changing the electromagnetic torque and thus the rotor’s speed^57–60^. By fine-tuning the generator’s operation to these calculated optimal points, the MPPT ensures that the wind turbine uses as much wind as possible to produce as much electricity as possible, improving the overall efficiency of the WECS. Moreover, the MPPT contributes to the grid-supporting capabilities of the system by ensuring that power is not only maximized but also stabilized, which is critical with the purpose of incorporating the produced power into the electrical system without causing fluctuations or disturbances.

The GSC’s role is pivotal in connecting the generator’s conditioned grid power. It also ensures power compatible with standard of grid, regulates both the voltage and frequency, and also manages reactive power to aid in grid stabilization. This regulation is essential for incorporating fluctuating grid system into reliable wind energy. Furthermore, system is equipped with filters on both rotor and grid sides to minimize electrical noise and harmonics, ensuring a smoother power flow into the grid. The overall control architecture aims to optimize the power output from wind while maintaining high-quality power and stability in the electrical grid^61–65^. This is achieved through a complex interplay of both the RSC and GSC, assisted by the advanced CSSO-ANFIS based MPPT and control algorithms. The efficacy of this intricate system is validated through rigorous simulations, which highlight its ability to maximize wind energy capture and concurrently support grid operations.

## System modelling

### Wind turbine modelling

Wind energy is transformed into torque, or rotational energy, by the wind turbine. (WT) is taken form the paper of this reference^24^. Equation (1) provides the wind’s power availability.

1 $$\:{P}_{v}=\frac{1}{2}\rho\:{AV}_{v}^{3}$$

where, $\:{V}_{v}$speed of wind$\:\left(m/\:s\right)$ A area that the turbine blades sweep$\:\left({m}^{2}\right)$,and ρ air density$\:\left(kg/{m}^{3}\right)$

Equation (2) provides the power that the turbine harvests from the available energy of the wind:

2 $$\:{P_t} = \frac{1}{2}\rho \:\pi \:{R^2}AV_v^3{C_p}\left( {\lambda ,\beta \:} \right)$$

Where $\:\left(\lambda,\beta\:\right)$pitch angle, *R* radius of rotor turbine$\:\left(r\right)$and $\:{C}_{p}\left(\lambda,\beta\:\right)$power of coefficient. $\:{C}_{p}$tip speed ratio function.

### DFIG modelling

**Fig. 2:**
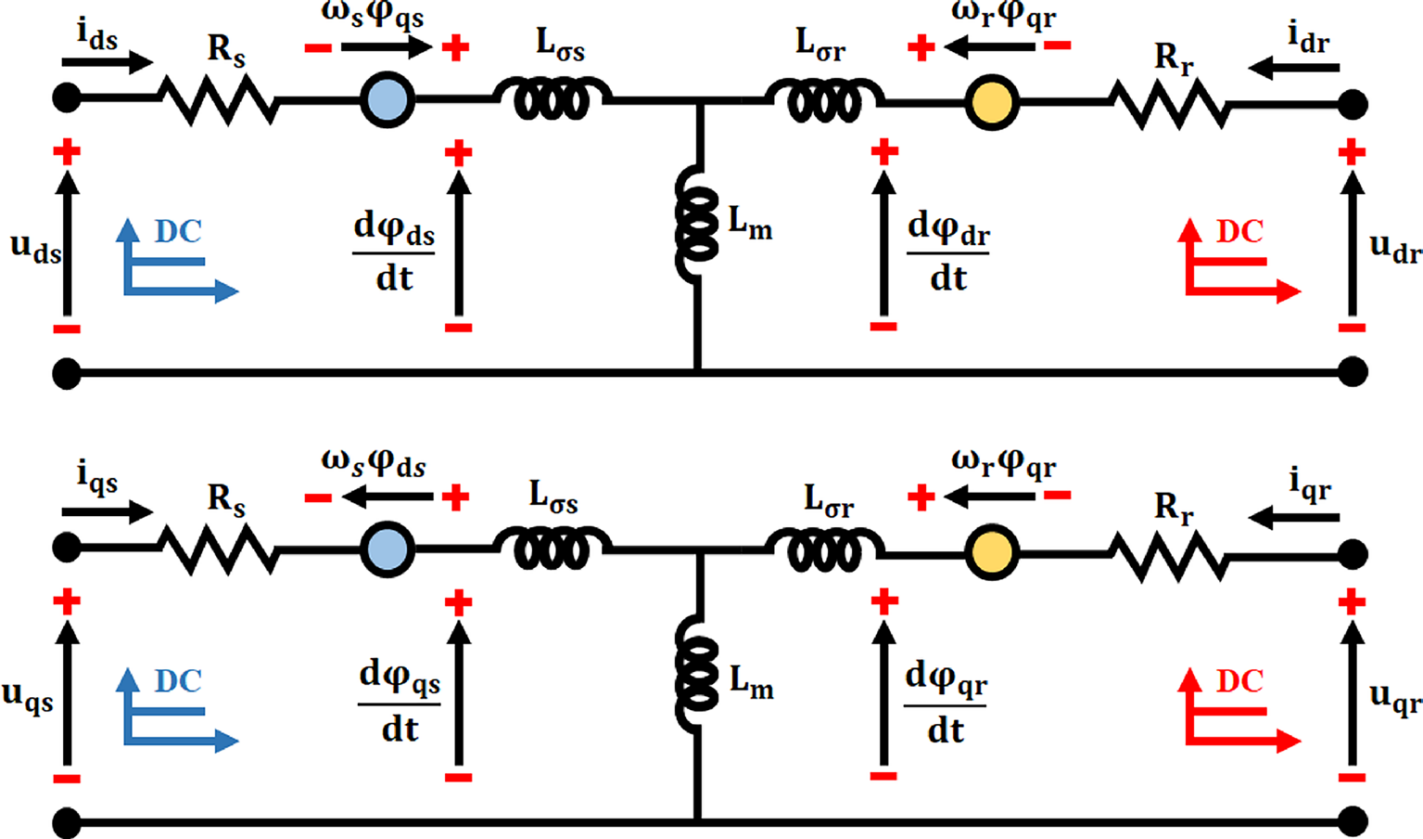
DFIG dq equivalent circuit.

The torque control modifies the rotor torque to get the intended power extraction, while the proposed MPPT control method controls the rotor speed to maintain the generator running at peak efficiency. The grid-side control loop integrates grid support operations by keeping an eye on energy transfer between the DFIG and the grid equations are taken from the paper of this reference^24^. Moreover, the RSC control approach, which also maintains in Fig. 2, the DC-link voltage regulates the power flow to the DFIG, and the GSC control technique regulates the power flow from the DFIG to the grid.

Transformations dynamic models are characterized using both direct and inverse methods. Two windings match the three windings of the rotor and stator using space vector theory (dq, revolving for the rotor, andαβ, stationary for the stator).

Between the voltage vector is represented by the stator and rotor as:

3 $$\:{\overrightarrow{u}}_{s}\Rightarrow\:\left\{\begin{array}{c}{u}_{ds}={R}_{s}{i}_{ds}+\frac{{d{\Psi\:}}_{ds}}{dt}-{w}_{s}{\psi\:}_{qs}\\\:{u}_{qs}={R}_{s}{i}_{qs}+\frac{{d{\Psi\:}}_{qs}}{dt}-{w}_{s}{\psi\:}_{ds}\end{array}\right.$$

4 $$\:{\overrightarrow{u}}_{s}\Rightarrow\:\left\{\begin{array}{c}{u}_{dr}={R}_{r}{i}_{dr}+\frac{{d{\Psi\:}}_{dr}}{dt}-{w}_{r}{\psi\:}_{qr}\\\:{u}_{qr}={R}_{r}{i}_{qr}+\frac{{d{\Psi\:}}_{qr}}{dt}-{w}_{r}{\psi\:}_{dr}\end{array}\right.$$

**Fig. 3:**
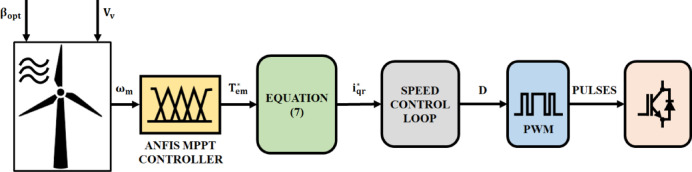
Schematic representation of ANFIS MPPT.

Hence, in the dq frame$\:{u}_{ds},{u}_{qs}{u}_{dr}$ and $\:{u}_{qr}$rotor and stator voltages, respectively. Stator, rotor current dq frame are denoted by the variables$\:{i}_{ds},{i}_{qs},{i}_{dr}$ and$\:{i}_{qr}$,respectively. $\:{R}_{r},{R}_{s},{w}_{s}$and$\:{w}_{r}$: the phase resistances and angular velocity of the stator and rotor, respectively. Figure 3 depicts the $\:dq$ equivalent electric circuit from Eqs. (3) and (4).

Equations (5) and (6) provide the expressions for the flux of stator and rotor vectors, respectively:

5 $$\:{\overrightarrow{\psi\:}}_{s}\Rightarrow\:\left\{\begin{array}{c}{\psi\:}_{ds}={L}_{s}{i}_{ds}+{L}_{m}{i}_{dr}\\\:{\psi\:}_{qs}={L}_{s}{i}_{qs}+{L}_{m}{i}_{qr}\end{array}\right.$$

6 $$\:{\overrightarrow{\psi\:}}_{r}\Rightarrow\:\left\{\begin{array}{c}{\psi\:}_{dr}={L}_{m}{i}_{ds}+{L}_{r}{i}_{dr}\\\:{\psi\:}_{qr}={L}_{m}{i}_{qs}+{L}_{r}{i}_{qr}\end{array}\right.$$

where, $$\:{\overrightarrow{\psi\:}}_{s}$$ the flow of stator and rotor vectors, respectively$$\:{\overrightarrow{\psi\:}}_{r}$$The stator’s dq axis fluxes are denoted by $$\:{\psi\:}_{ds}$$ and$$\:{\psi\:}_{qs}$$Together with the dq axis rotor, the fluxes are $$\:{\psi\:}_{dr}$$ and$$\:{\psi\:}_{qr}$$$$\:{L}_{s}$$$$\:{L}_{r}$$ stand for the phase rotor and stator leakage inductances, respectively. $$\:{L}_{m}$$ Stands for stator-rotor mutual inductance, while $$\:p$$ denotes the generator’s number of pole pairs.By figuring out the ideal operating conditions, DFIG and ANFIS combined with MPPT enable the wind turbine to produce more electricity more efficiently.

(7) is the expression for electromagnetic torque.

7 $$\:{T}_{em}=\frac{3}{2}p\frac{{L}_{m}}{{L}_{s}}\left({\psi\:}_{qs}{i}_{dr}-{\psi\:}_{ds}{i}_{qr}\right)$$

(8) and (9) display the stator and rotor active and reactive power Eq. 

8 $$\:\left\{\begin{array}{c}{P}_{s}=\frac{3}{2}\left({u}_{ds}{i}_{ds}+{u}_{qs}{i}_{qs}\right)\\\:{Q}_{s}=\frac{3}{2}\left({u}_{qs}{i}_{ds}-{u}_{ds}{i}_{qs}\right)\end{array}\right.$$

9 $$\:\left\{\begin{array}{c}{P}_{r}=\frac{3}{2}\left({u}_{dr}{i}_{dr}+{u}_{qr}{i}_{qr}\right)\\\:{Q}_{r}=\frac{3}{2}\left({u}_{qr}{i}_{dr}-{u}_{dr}{i}_{qr}\right)\end{array}\right.$$

where The stator’s active and reactive power are shown by$\:{P}_{s}$ and$\:{Q}_{s}$. $\:{P}_{r}$, $\:{Q}_{r}$ displays an active rotor and power in reaction. Electromagnetic torque is measured in$\:{T}_{em}$.

### ANFIS MPPT

The Adaptive neuro-fuzzy inference method of the following reference is taken of from this paper^25^, which combines artificial neural networks and fuzzy control concepts, is highly effective. It excels as a translator and learner due to the combined effects of fuzzy logic and neural networks. The membership function to be used is chosen by the ANFIS controller^43,66–69^. Five tiers make up the overall design of ANFIS.

- Membership functions (MF) and user-specified input variables make up Layer 1 is the adaptive fuzzification layer.
- The fuzzy rule layer in layer 2 determines the degree of membership function (MF), selecting the matching fuzzy set to be passed layer next.
- Each normalized node’s weight is assessed by the third layer, which also normalizes dismissal strength.
- Each neuron is normalized by the adaptive implication layer, or layer 4, which produces values by applying the inference principles.
- Fuzzy values are converted into crisp values by Layer 5’s output layer by summing up all of the inputs from Layer 4.

Rotor speed is the only input used by the developed ANFIS. The output of the ANFIS network is used to calculate the instantaneous torque reference. The Takagi and Sugeno fuzzy IF-THEN rules and the ANFIS first-order Sugeno model types are both utilized in the created MPPT controller. The MPPT controller based on ANFIS is trained via a backpropagation technique.

Figure 3 shows the proposed ANFIS MPPT control block diagram. RSC control’s speed control loop receives the rotor quadrature current reference$\:\left({i}_{qr}^{*}\right)$. Based on the generated optimal torque$\:\left({T}_{qr}^{*}\right)$, the RSC control modifies RSC duty ratio to modify real rotor speed. Optimizing the amount of output power sent to the grid is the converter’s control task.

**Fig. 4:**
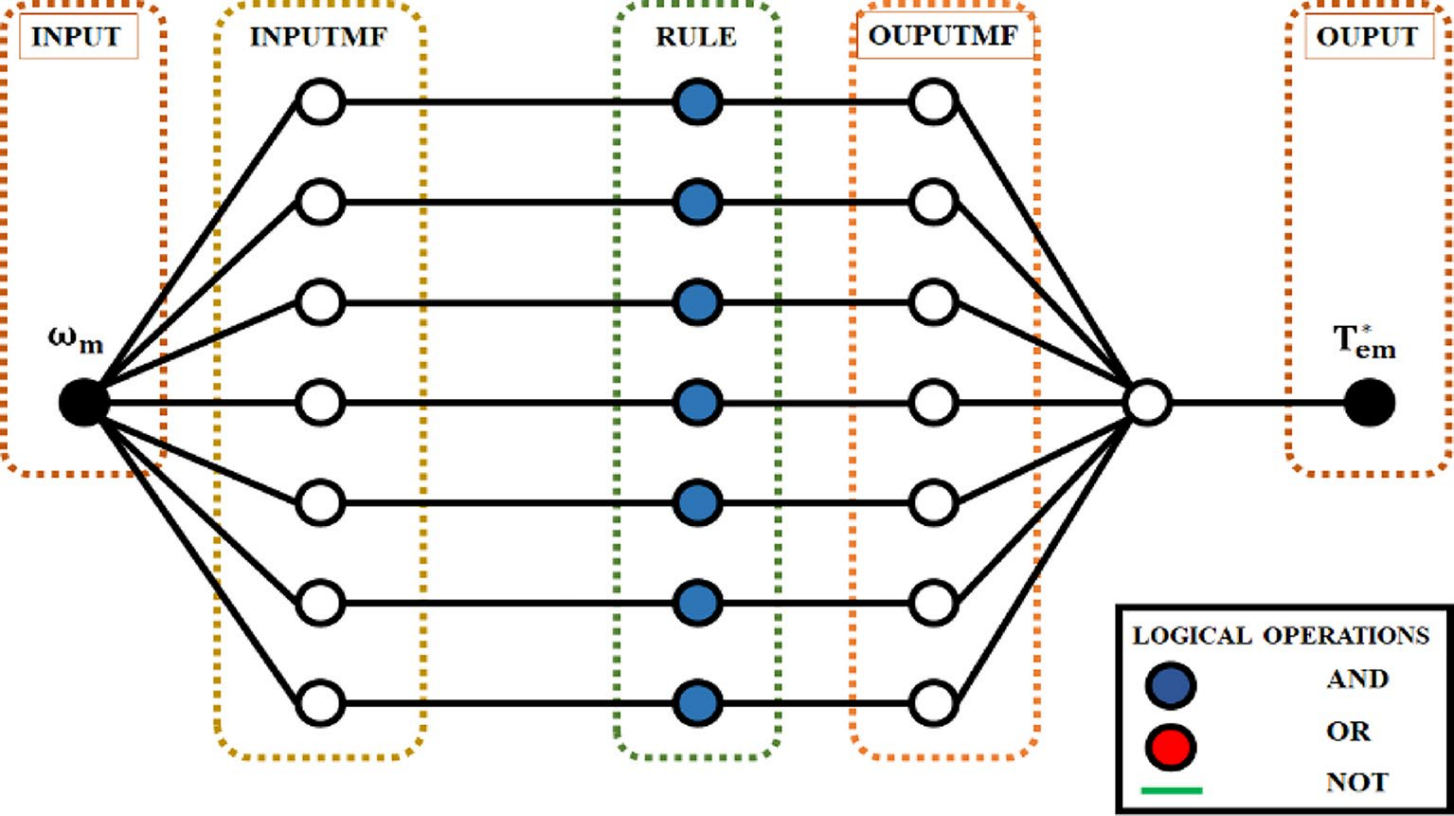
The architecture of the ANFIS controller.

The ANFIS controller’s architecture, created using Neuro-Fuzzy Designer is shown in Fig. 4 in MATLAB/Simulink. Quantity and shape of membership functions (MFs) are chosen by iterative testing, since the literature currently in publication does not provide a clear approach for choosing MFs.

In a fuzzy logic system, rules define the relationships between inputs and outputs, input variables are fuzzified into fuzzy sets using membership functions, and fuzzy sets are manipulated logically using operations like AND, OR, and NOT to control systems or make decisions.

### Chaotic salp swarm algorithm

Several advantages shared by advantages of Simpleness, scalability, and the ability to shorten calculation times are advantages of population-based meta-heuristic algorithms. The two primary drawbacks of these algorithms are their low convergence rate and recession in local optima. Using chaos theory is one method to get around these issues and improve the efficiency of meta-heuristic algorithms. PSO-based algorithms employ chaotic maps rather than random numbers to improve convergence.

As a result, the authors offer an SSA that is chaotic (CSSA), substituting chaotic variables for random ones. The CSSO is designed for optimizing complex systems by the integration of chaos theory. The standard SSA mimics the swarm behaviour of salps, with a leading salp guiding followers through an exploration-exploitation process to find optimal solutions. However, traditional SSA suffers from slow convergence rates and a tendency to become trapped in local optima. CSSO addresses these issues by replacing random variables with chaotic maps, such as the logistic map, which introduces deterministic yet unpredictable behaviour into the optimization process. This integration of chaos enhances population diversity, improves convergence rates and prevents premature stagnation. The CSSA modifies using chaotic maps to calculate the second coefficient’s value,$\:{C}_{2}$As seen in the example below, The significance of a workable chaotic map is substituted for $\:{C}_{2}$in the current iteration

10 $$\:{C}_{2}^{t}=\omega\:\left(t\right)$$

And chaotic map value of iteration is$\:{t}^{th}$denoted by$\:\omega\:\left(t\right)$.With the updated value of$\:{C}_{2}$the equation may be rewritten as follows

11 $$\:{X}_{i}^{1}=\left\{\begin{array}{c}{F}_{i}+{C}_{1}\left(\left({ub}_{i}-{ul}_{i}\right)\omega\:\left(t\right)+{lb}_{i}\right),\:{C}_{3}\ge\:0\\\:{F}_{i}+{C}_{1}\left(\left({ub}_{i}-{ul}_{i}\right)\omega\:\left(t\right)+{lb}_{i}\right),\:{C}_{3}<0\end{array}\right.$$

The ANFIS model’s parameters are adjusted using CSSO using real-time wind turbine data, including generator and wind speed. The technology can follow the maximum power point more precisely and maximize the wind turbine’s energy output by fine-tuning the ANFIS parameters.

### Chaotic maps

A popular mathematical method for examining the behaviour of dynamic systems with crucial beginning conditions is chaos theory. Using discrete or continuous chaotic maps is one method to illustrate this phenomenon. The use of chaotic maps is limited to deterministic systems with predictable actions. Recently, numerous systems in diverse domains like computer science, robotics, physics, and microbiology have grown more interested in chaos theory.

When it comes to optimizing the randomization parameters of meta-heuristic algorithms, chaotic maps prove to be the most potent approach. These arbitrary parameters are taken out and founded on a Gaussian or uniform distribution, making them more controllable by a chaotic map that exhibits the same quality but performs better. By adjusting these variables with chaotic maps, the convergence is increased and the number of local optima is decreased.

The logistic map is a best chaotic map for our optimizer based on the outcomes:

12 $$\:\omega\:\left(t+1\right)=a\omega\:\left(t\right)\left[1-\omega\:\left(t\right)\right],\:a=4$$

And the chaotic map value at the $\:{t}^{th}$ iteration is denoted by$\:\omega\:\left(t\right)$ It is assumed that the chaotic map’s initial state is $\:0.7\:\left(\omega\:\right(0)\:=\:0.7).$

The pseudocode of the proposed CSSO is defined at algorithm 1.

**Algorithm 1:**
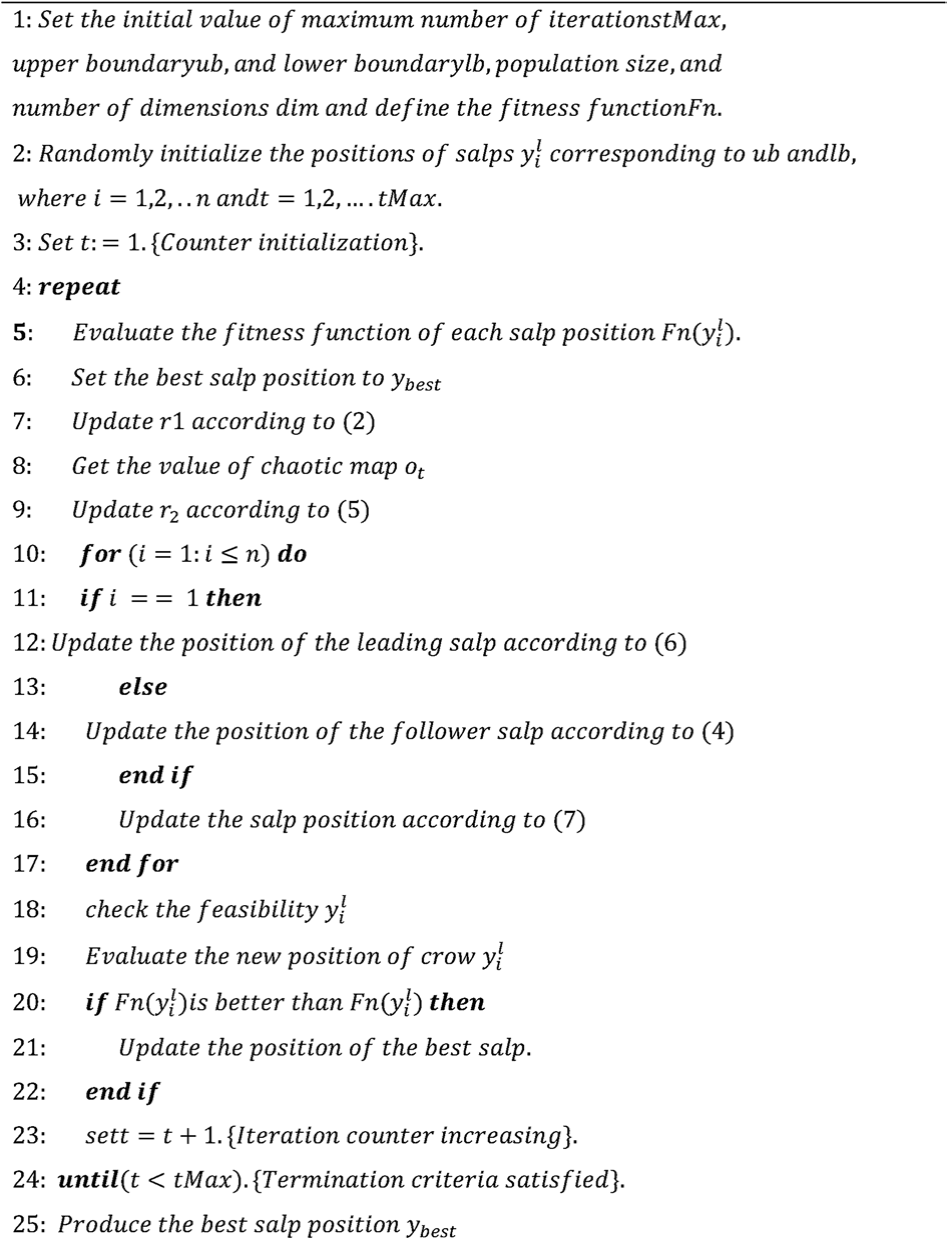
Pseudocode of CSSO algorithm.

The integration of the CSSO algorithm with ANFIS for MPPT in wind energy systems enhances the efficiency and robustness of the MPPT solution. The parameters of the ANFIS model are adjusted to improve the MPPT efficiency. The membership function parameters, rule weights and coefficients are tuned by CSSO. The parameters are randomly initialized within the defined bounds. Based on the energy extraction efficiency, the fitness of the parameter set is evaluated by CSSO. Using the fitness function, the position of each salp is evaluated. The ANFIS parameters are dynamically tuned by CSSO depending on wind turbine data thereby adapting to varying environmental conditions. In each iteration, CSSO modifies the ANFIS parameters and evaluates their impact on MPPT performance. CSSO’s chaotic search strategy improves exploration and exploitation, leading to quicker convergence to the global optimum. ANFIS is adept at modelling complex nonlinear relationships, making it well-suited for capturing the nuances of wind energy systems. This combined approach enables better adaptation to changing environmental conditions and enhances overall system performance, resulting in higher energy extraction and efficiency.

### Control strategy for RSC and GSC

The DFIG stator winding is supplied with a constant magnitude and frequency of three-phase grid power. On the other hand, RSC supplies the rotor at various magnitudes and frequencies to achieve various DFIG working conditions. The machine’s operating point determines how much power goes via the grid and the rotor is referred form this paper^24^. Equation (11) provides the speed at which each of the three DFIG working modes is dependent.

13 $$\:\left\{\begin{array}{c}{{\upomega\:}}_{\text{S}}={{\upomega\:}}_{\text{r}}+{{\upomega\:}}_{\text{m}}\\\:s=\frac{{{\upomega\:}}_{\text{S}}-{{\upomega\:}}_{\text{m}}}{{{\upomega\:}}_{\text{S}}}\end{array}\right.$$

14 $$\:\left\{\begin{array}{c}{{\upomega\:}}_{\text{m}}<{{\upomega\:}}_{\text{S}}\Rightarrow\:{{\upomega\:}}_{\text{S}}>0\Rightarrow\:s>0\Rightarrow\:Subsynchronous\:operation\\\:{{\upomega\:}}_{\text{m}}>{{\upomega\:}}_{\text{S}}\Rightarrow\:{{\upomega\:}}_{\text{r}}<0\Rightarrow\:s<0\Rightarrow\:Hypersynchronous\:operation\\\:{{\upomega\:}}_{\text{m}}={{\upomega\:}}_{\text{S}}\Rightarrow\:{{\upomega\:}}_{\text{r}}=0\Rightarrow\:s=0\Rightarrow\:Synchronous\:operation\end{array}\right.$$

DFIG $\:dq$ frame, vector control approach is applied. This causes the $\:d$ and $\:q$ values to spontaneously decouple. When disconnected, serves as the d axis of motor DC, along which the stator flux vector is driven.

(i) Rotor side control At rotor winding, the voltage is applied by the RSC. Equations (5) and (6)and be substituted into Eq. (4) to obtain voltage equations in the dq frame. Taking $\:\:{\psi\:}_{qs}=\:0$ into consideration results in the voltage expressions that follow:

15 $$\:\left\{\begin{array}{c}{u}_{dr}={R}_{r}{i}_{dr}+\sigma\:{L}_{r}\frac{{di}_{dr}}{dt}-{{\upomega\:}}_{\text{r}}\sigma\:{L}_{r}{i}_{qr}+\frac{{L}_{m}}{{L}_{s}}\frac{{\overrightarrow{d\psi\:}}_{s}}{dt}\\\:{u}_{qr}={R}_{r}{i}_{qr}+\sigma\:{L}_{r}\frac{{di}_{qr}}{dt}-{{\upomega\:}}_{\text{r}}\sigma\:{L}_{r}{i}_{qr}+{{\upomega\:}}_{\text{r}}\frac{{L}_{m}}{{L}_{s}}\frac{{\overrightarrow{d\psi\:}}_{s}}{dt}\end{array}\right.$$

where,$\:\sigma\:=1-{L}_{m}^{2}/{L}_{s}{L}_{r}$the stator winding resistance to decrease and the stator flux to be regarded as constant be disregarded because of the fixed grid values $\:\frac{{\overrightarrow{d\psi\:}}_{s}}{dt}$ close to zero. Equation (15) makes it clear that the regulators enabled to regulate the rotor current’s dq component.

(ii) Grid Side Control.

**Fig. 5:**
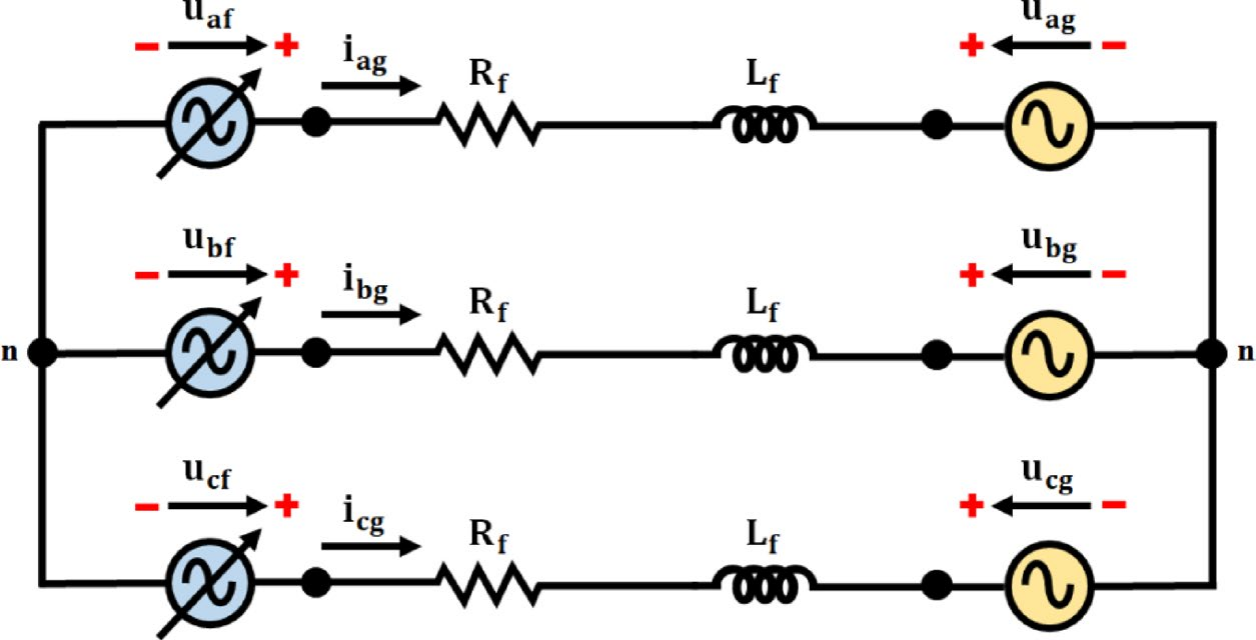
A simplified diagram illustrating the three-phase grid system.

Figure 5 depicts the system configuration made up of the grid voltage, filter, and GSC. The DFIG’s power flow is managed by the GSC control technique.

When controlling power flow, it is crucial to consider two key factors: the DC link voltage $\:{V}_{bus}$and the exchange of reactive power $\:{Q}_{g}$ with the grid.

16 $$\:\left\{\begin{array}{c}{u}_{dg=\left|{\overrightarrow{u}}_{g}\right|}\\\:{u}_{qg=0}\\\:{\omega\:}_{a}={\omega\:}_{s}\\\:\theta\:={\omega\:}_{a}t\Rightarrow\:\theta\:={\theta\:}_{g}={\omega\:}_{s}t\end{array}\right.$$

17 $$\:\left\{\begin{array}{c}{u}_{df}={R}_{f}{i}_{dg}+{L}_{f}\frac{{di}_{dg}}{dt}+{u}_{dg}-{\omega\:}_{s}{L}_{f}{i}_{qg}\\\:{u}_{qf}={R}_{f}{i}_{qg}+{L}_{f}\frac{{di}_{qg}}{dt}+{\omega\:}_{s}{L}_{f}{i}_{dg}\end{array}\right.$$

Equation (16), which describes the voltage vector’s direct component, is aligned with$\:\omega\:s$. Equation (17) expresses the interchange of active and reactive power with the grid and determines the dq component of the filter voltage.

## Result and discussion

The proposed control method for a WECS-DFIG system, validated through MATLAB/Simulink, effectively adjusts rotor speed and torque for optimal power extraction, regulates power flow between DFIG and grid, and provides grid support functions like voltage regulation and harmonic mitigation. The simulations demonstrate its potential to improve wind energy systems’ efficiency.

**Table 2:** Parameter specification.

Simulation parameters			
Parameter	Specification	Parameter	Specification
No. of Turbines	$\:4$	Nominal wind speed	$\:11m/s$
Inertia Constant	$\:0.685Hs$	Air Density	$\:1.225\:kg/{m}^{3}$
Pairs of Poles	$\:3$	Tip speed ratio	7
Friction Factor	$\:0.01F\left(pu\right)$	Pitch angle	$\:{0}^{^\circ\:}$
Magnetizing Inductance	$\:2.9mH$	Power coefficient	0.4411
Rotor Inductance	$\:0.16mH\left(pu\right)$	Nominal power	$\:2\:MW$
Rotor Resistance	$\:0.016\varOmega\:\:\left(pu\right)$	Frequency	$\:50\:Hz$
Stator Resistance	$\:0.023\:\varOmega\:\:\left(pu\right)$	Rated Torque	$\:\text{12,732}N\bullet\:m$
Stator Inductance	$\:0.18mH\:\left(pu\right)$	Pole Pair	$\:2$
Frequency	$\:50Hz$	Inertia	$\:127\:kg\bullet\:{m}^{2}$
Line-Line Voltage	$\:415V$	Gear ratio	$\:100$
Nominal Power	$\:10kW$	Radius of turbine	$\:42m$

### Case 1-constant wind speed

**Fig. 6:**
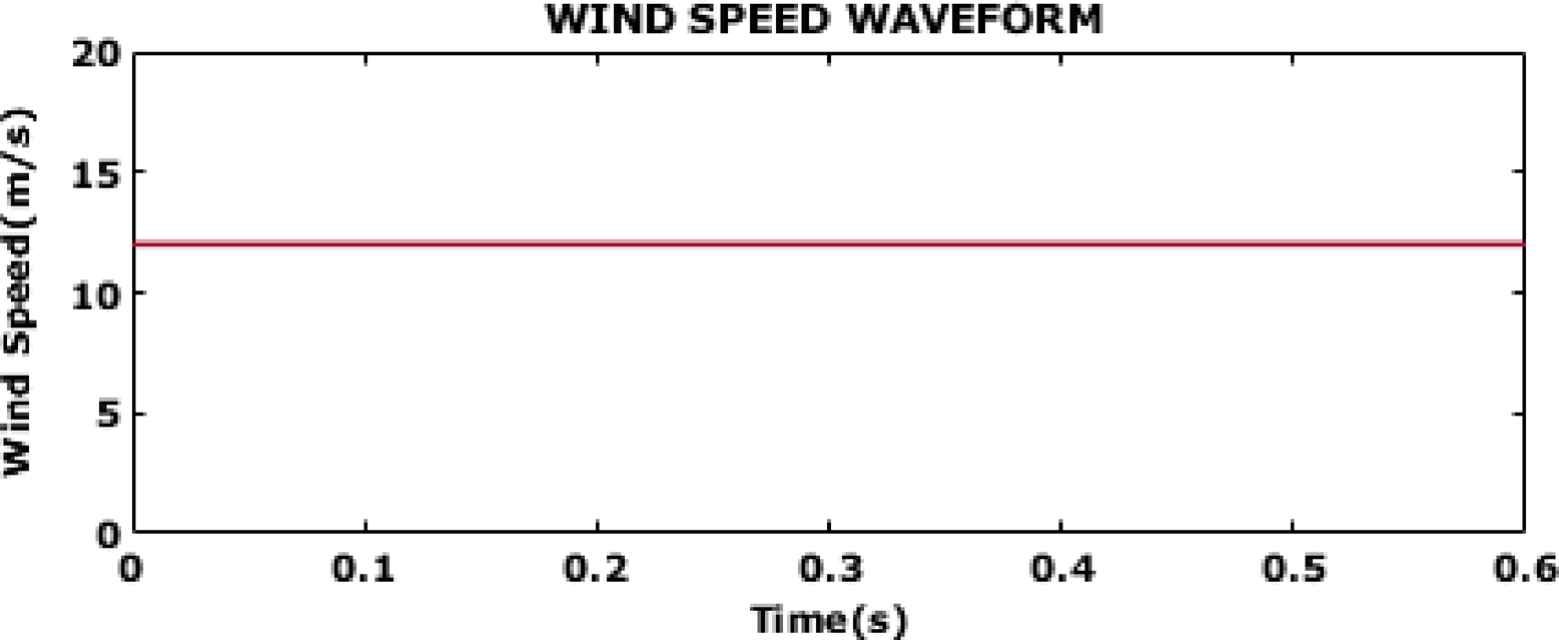
Wind speed waveform.

Figure 6 depicts the wind speed over time with particular focus on a constant speed of 12 m/s. Because it offers a consistent and dependable source of wind power a requirement for wind turbines to operate efficiently this steady state is perfect for the production of wind energy.

**Fig. 7:**
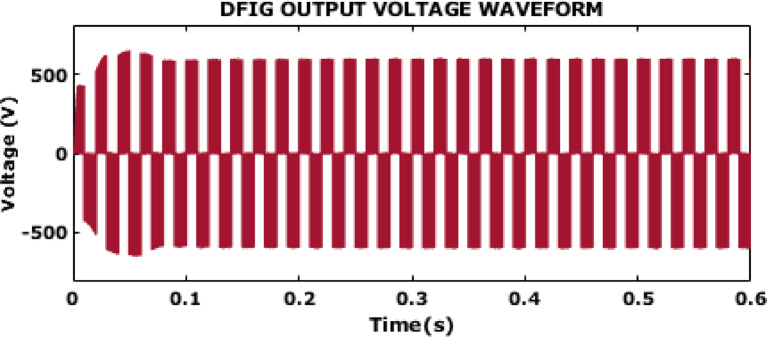
DFIG voltage waveform output.

Figure 7 represents a DFIG output voltage waveform that has a stable 600 V supply voltage after $\:0.1s.$ For the DFIG in a wind energy system to be dependable and efficient, this steady voltage output is essential. At the start of the simulation, there is a transient response, where the voltage varies significantly before stabilizing. After this initial period, the output voltage waveform becomes more consistent, oscillating around a peak of approximately ± 600 V. The smooth and consistent oscillations in the steady state imply that the system achieves effective control of voltage and current harmonics, ensuring reliable and stable power delivery to the grid.

**Fig. 8:**
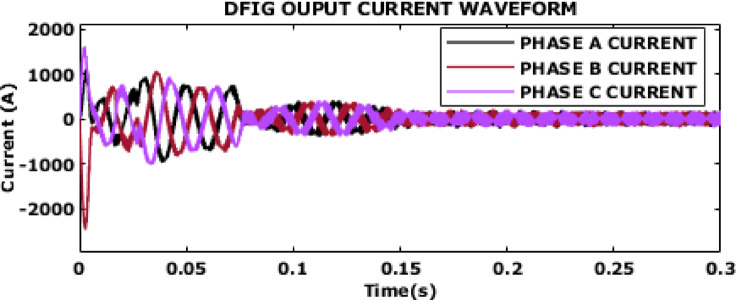
DFIG output current waveform.

The DFIG output current waveform with peak current of 67 A after 0.15s is provided in Fig. 8. Initially, all the three phase currents exhibit high oscillations and large peak values as the system adjusts to operating conditions. The current begins to stabilize and settle into a more consistent oscillatory pattern at 0.15s. The stabilization of the current waveform shows the effectiveness of the control strategy in handling transients and achieving stable performance. The steady-state current waveform’s consistency also highlights the control system’s ability to mitigate harmonic distortions and ensure smooth power delivery, which is essential for grid-connected operations.

**Fig. 9:**
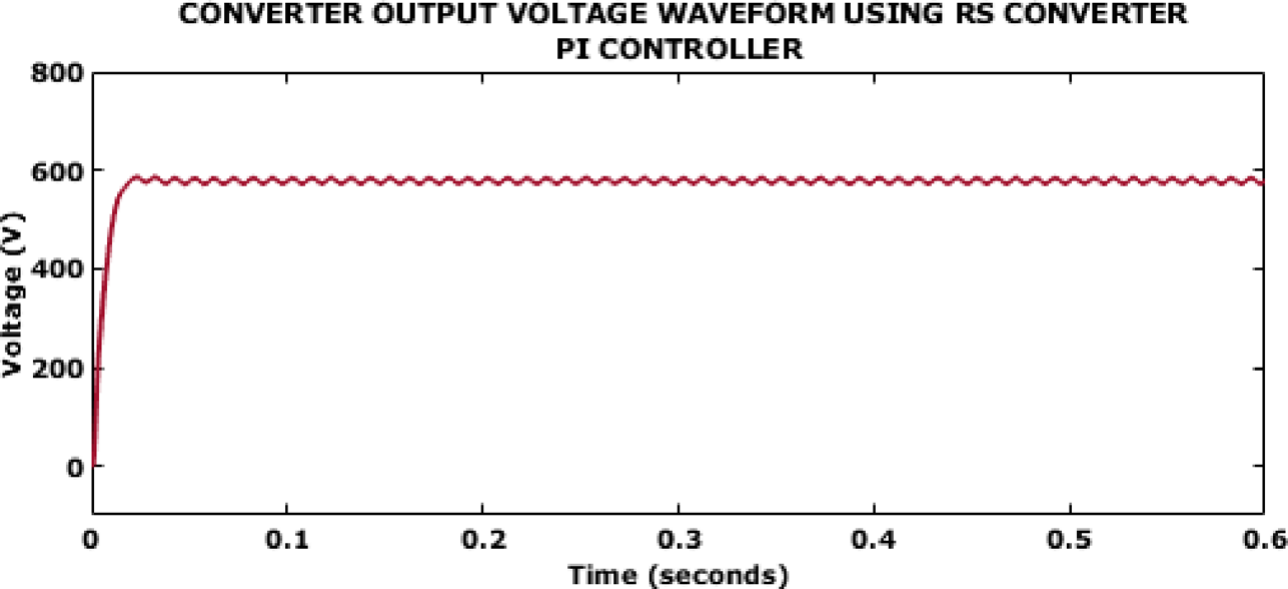
Converter output voltage waveform using RS converter PI controller.

Figure 9 represents the output voltage attained using the PI controller. The attained voltage is around 600 V but the outcome gets stabilized only after 0.6s.

**Fig. 10:**
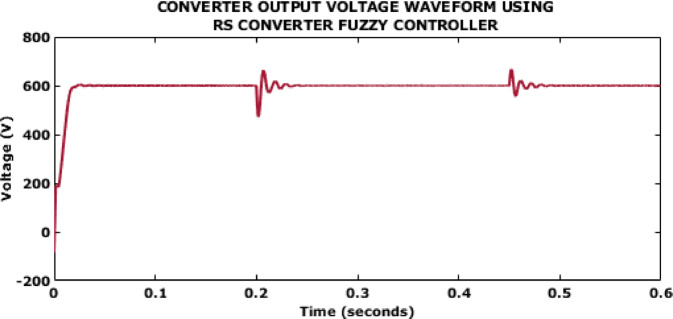
Converter output voltage waveform using RS converter Fuzzy controller.

Figure 10 illustrates the converter output using Fuzzy controller which indicates a value of 600 V. This control aids in the generation of a settling time of 0.5s which could be reduced further for improved stability.

**Fig. 11:**
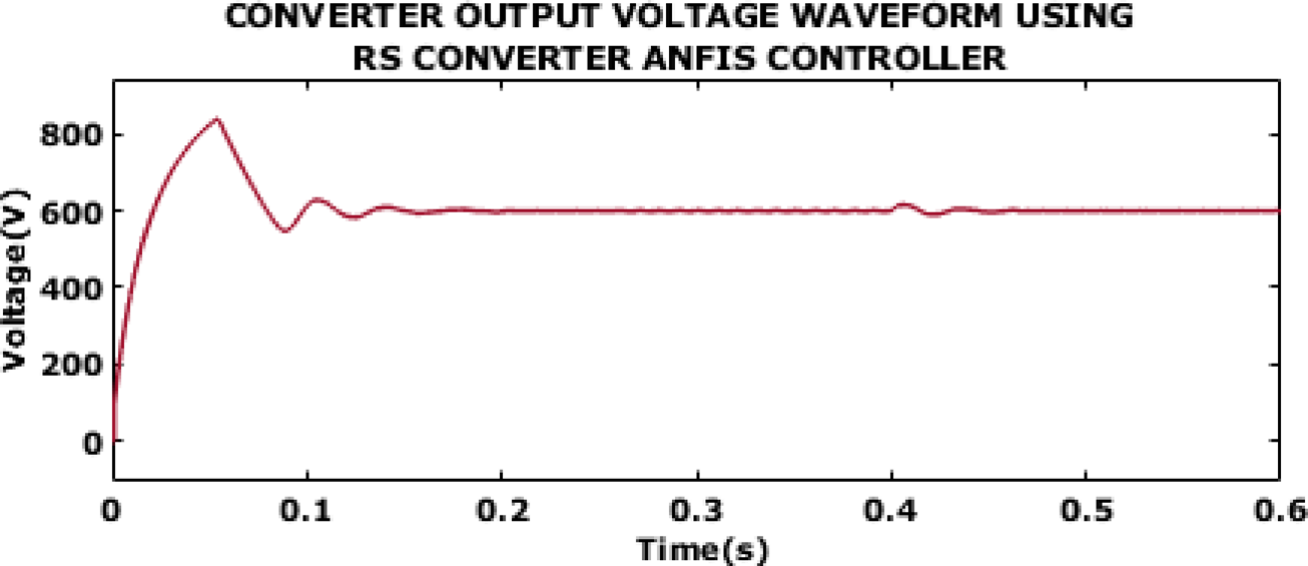
Converter output voltage waveform using RS converter ANFIS controller.

The output voltage waveform of converter with ANFIS controller is displayed in Fig. 11. The waveform shows high initial overshoot, which in turn leads to stress on the converter. Moreover, the voltage experiences prolonged settling time and shows oscillatory behaviour before stabilizing at 0.46s.

**Fig. 12:**
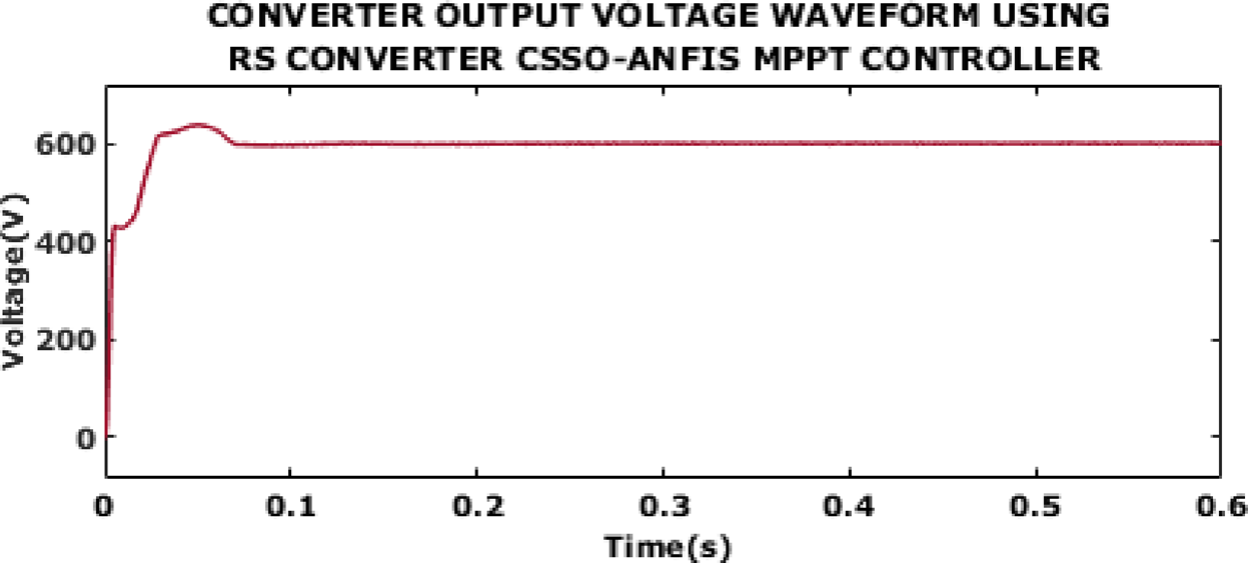
Converter output voltage waveform using RS converter CSSO-ANFIS MPPT controller.

A converter output voltage waveform using RS converter with CSSO-ANFIS MPPT is in Fig. 12. The proposed control ensures rapid response, minimal overshoot and fast stabilization. After the initial adjustment, the converter output voltage exhibits a stable value of 600 V at 0.13s with almost no oscillations or ripples.

### Case 2- varying wind speed

**Fig. 13:**
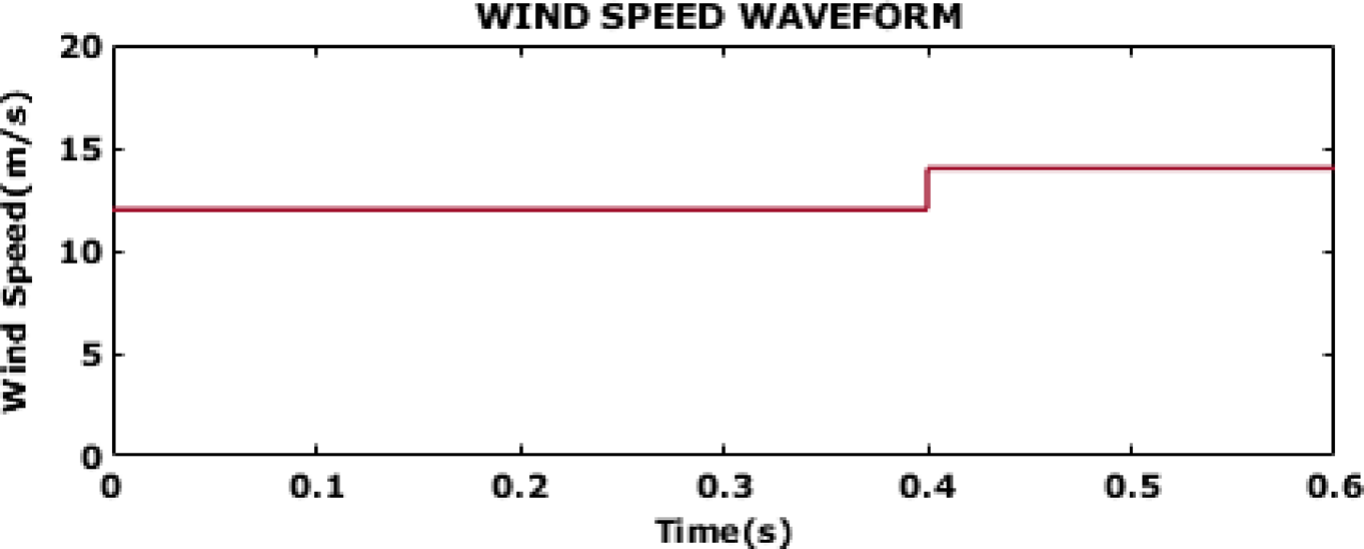
Wind speed waveform.

The wind speed waveform for case 2 is depicted on a waveform in Fig. 13. In case 2, the proposed control is tested for variable wind speed. For the first 0.4 s of the simulation, the wind speed is maintained at a constant 12 m/s. At around 0.4 s, there is a sudden increase in wind speed from 12 m/s to 14 m/s. This step change in wind speed introduces a dynamic change in the system and the impacts are further evaluated by CSSO-ANFIS.

**Fig. 14:**
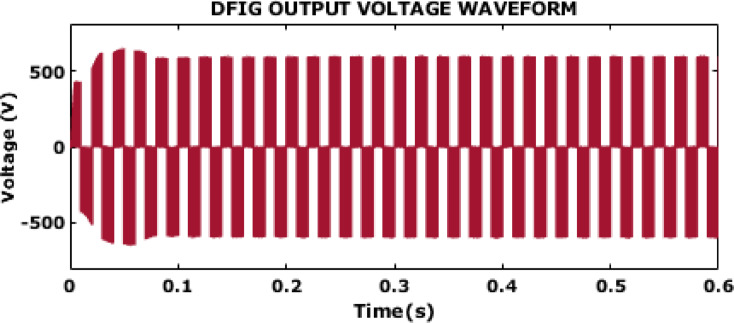
DFIG output voltage waveform.

The DFIG output voltage waveform for case 2 is provided in Fig. 14. Initially, the waveform shows some oscillations and instability, particularly in the first 0.1 s, with a significant variation in voltage amplitude. This transient behaviour is expected as the DFIG adjusts to the initial conditions and begins to stabilize. After this initial period, the voltage waveform settles into a more consistent oscillatory pattern around a peak value of approximately ± 600 V. In spite of varying wind conditions, the DFIG generates a stabilized output with the adopting of the proposed control.

**Fig. 15:**
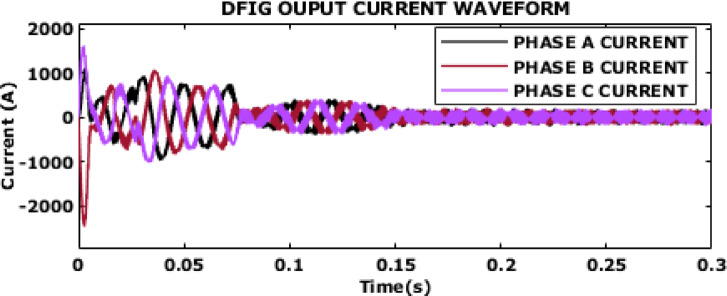
DFIG output current waveform.

DFIG’s output current waveform is shown in Fig. 15. After initial transient response, the oscillations in current waveform begin to dampen, and it transitions into a more stable pattern. The observed damping and stabilization suggest that the DFIG effectively manages the initial transients and quickly stabilizes the current output. This quick stabilization highlights the robustness of the system in handling transient conditions and achieving improved performance thereby assuring reliable current delivery.

**Fig. 16:**
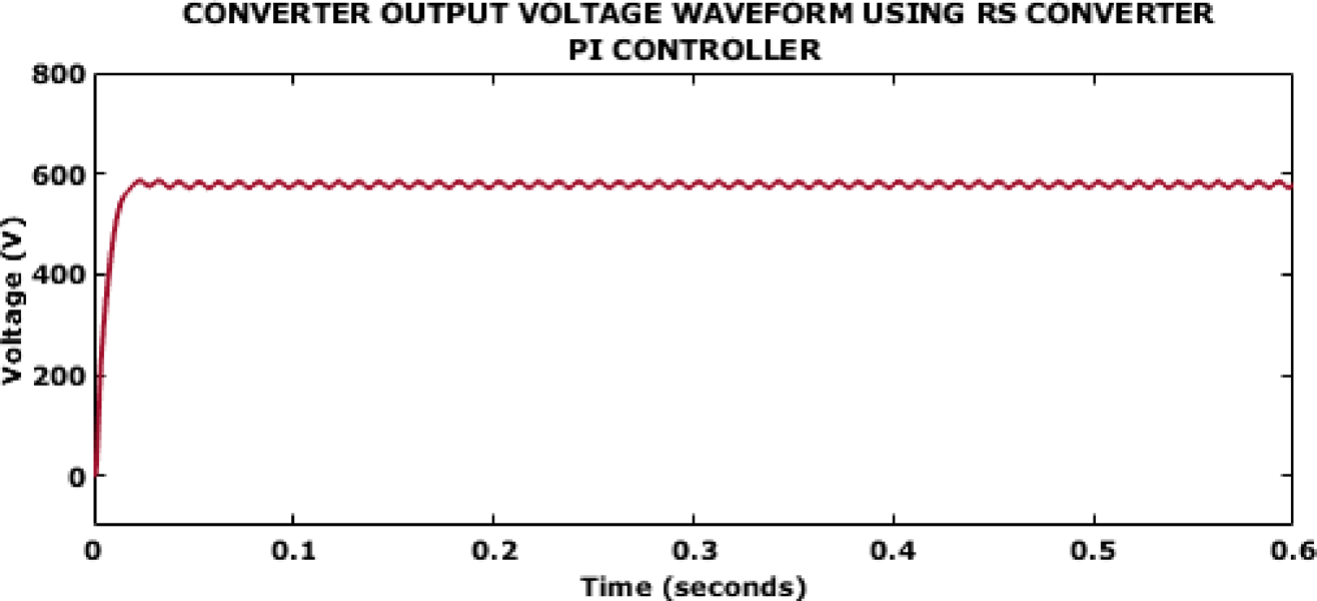
Converter output voltage waveform using RS converter PI controller.

The converter output achieved for case 2 using conventional PI controller is shown in Fig. 16. An output of 600 V is attained in this varying wind speed condition but takes longer time to stabilize almost greater than 0.6s.

**Fig. 17:**
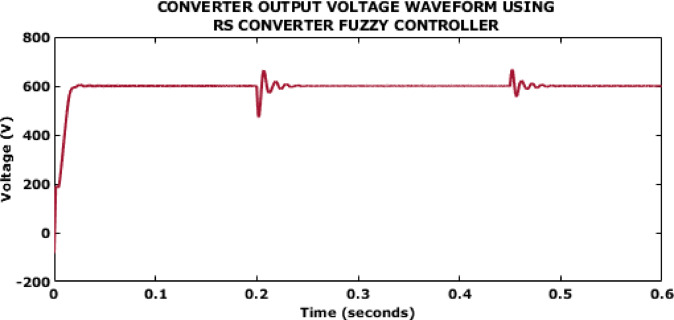
Converter output voltage waveform using RS converter Fuzzy controller.

Figure 17 denotes the obtained output for converter using Fuzzy controller in which the voltage indicates a value of 600 V. The output gets stabilized at a settling time of 0.5s which needs further improvement.

**Fig. 18:**
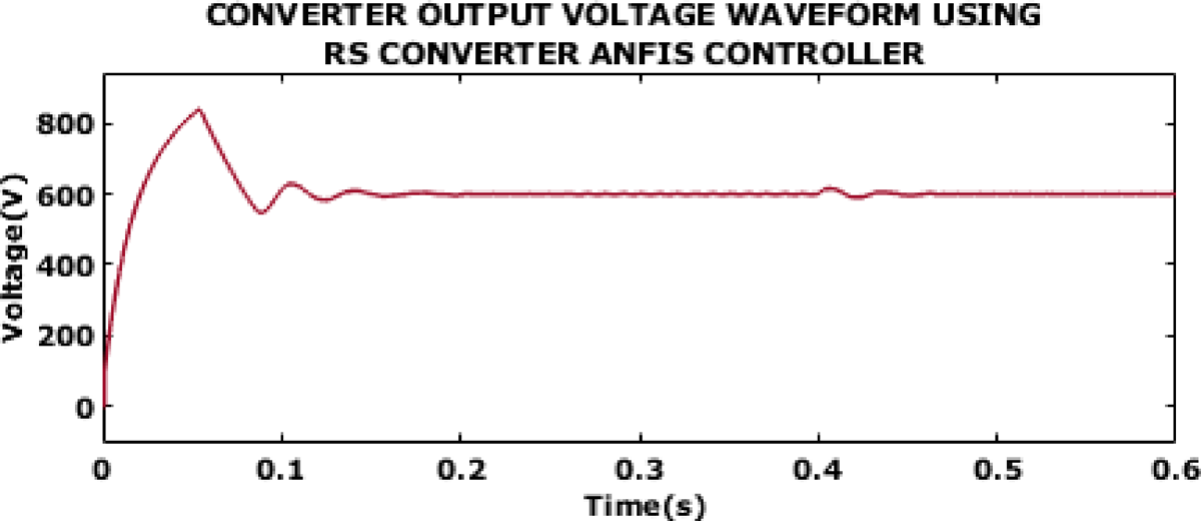
Converter output voltage waveform using RS converter ANFIS controller.

Figure 18 shows the output voltage waveform of converter using ANFIS controller. The waveform shows significant initial overshoot and oscillatory behaviour and a longer time to stabilize at around 0.48s. Additionally, the presence of steady-state ripples causes power quality issues. While ANFIS achieves eventual stabilization, optimizing the control parameters could improve transient response, reduce ripples and enhance overall power quality.

**Fig. 19:**
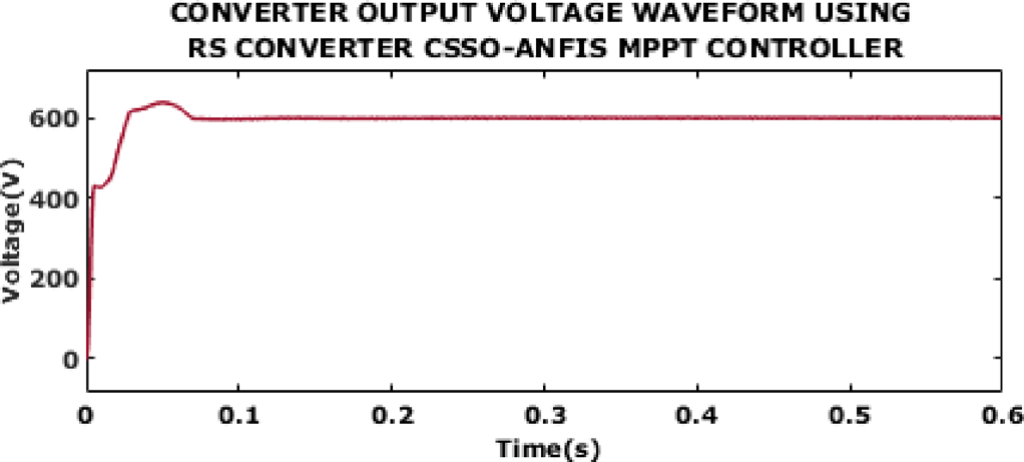
Converter output voltage waveform using RS converter CSSO-ANFIS MPPT controller.

The output voltage waveform of RS converter with CSSO-ANFIS MPPT is shown in Fig. 19. The stability observed in the voltage waveform indicating a value of 0.13s demonstrates the effectiveness of the controller in maintaining a consistent and high-quality voltage output despite the variations in wind speed. This performance proves the controller’s efficacy for ensuring reliable and efficient operation of the DFIG-based WECS, with minimal impact on power quality and grid compatibility.

**Fig. 20:**
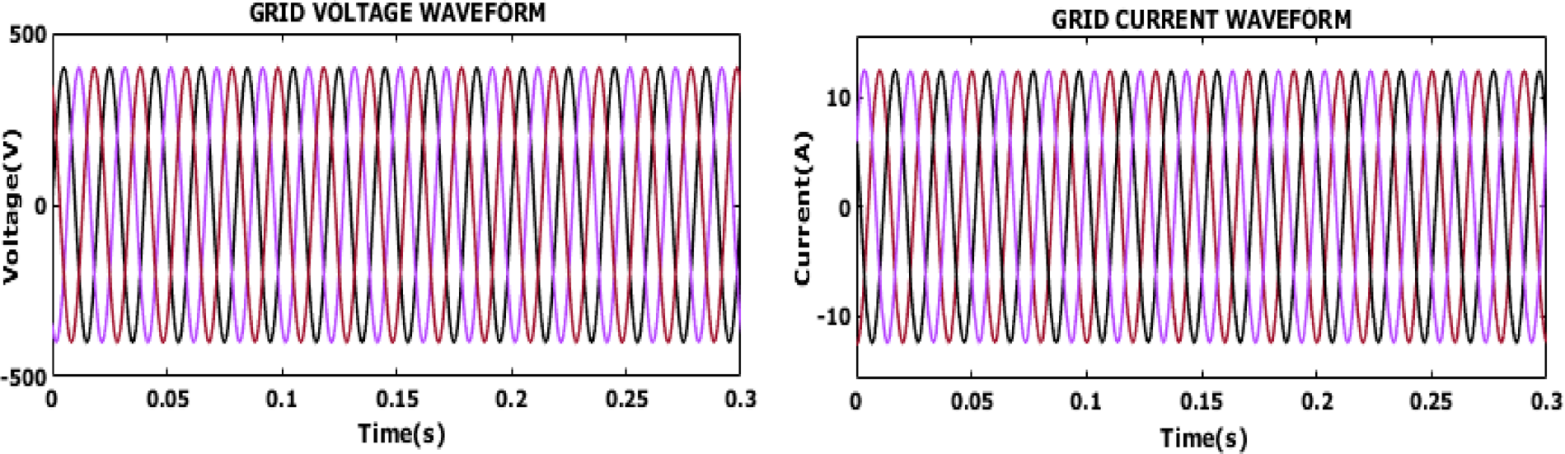
Grid voltage and current waveform.

Figure 20 displays the grid voltage and current outcomes. The consistent and sinusoidal nature of both the voltage and current waveforms indicates that the system is effectively managing power delivery to the grid, ensuring stable and efficient operation. The results confirm that the implemented control strategy efficiently synchronizes the system with the grid to facilitate smooth and reliable energy transfer, even under variable operating conditions.

**Fig. 21:**
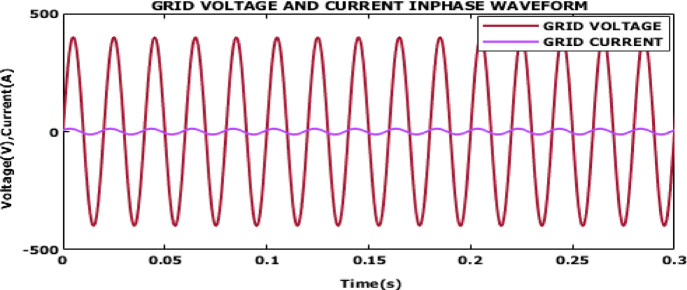
Grid voltage and current waveform.

The waveform in Fig. 21 shows a single-phase of both grid voltage and current over a period of 0.3 s. The voltage and current waveforms are in phase with each other indicating improved power quality.

**Fig. 22:**
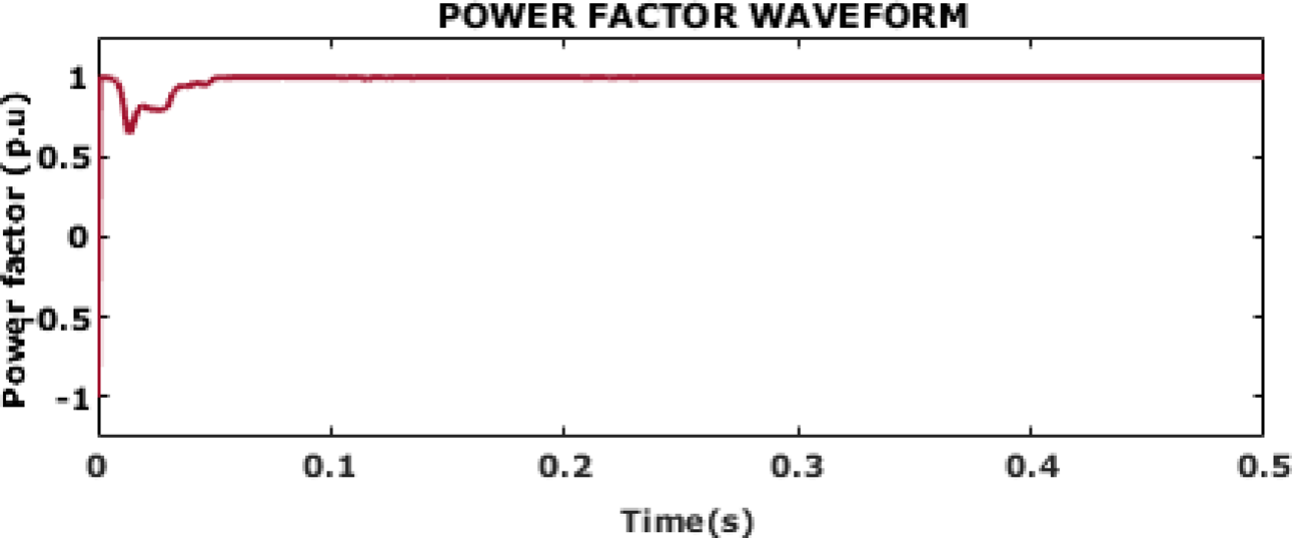
Power factor waveform.

The waveform in Fig. 22 illustrates the power factor waveform. The stable near unity power factor indicates that the system is operating at optimal efficiency, ensuring that the maximum amount of power is being delivered to the grid without losses associated with reactive power. The power factor output confirms that the suggested control is highly suited for grid integration providing high power quality and reliable delivery of energy.

**Fig. 23:**
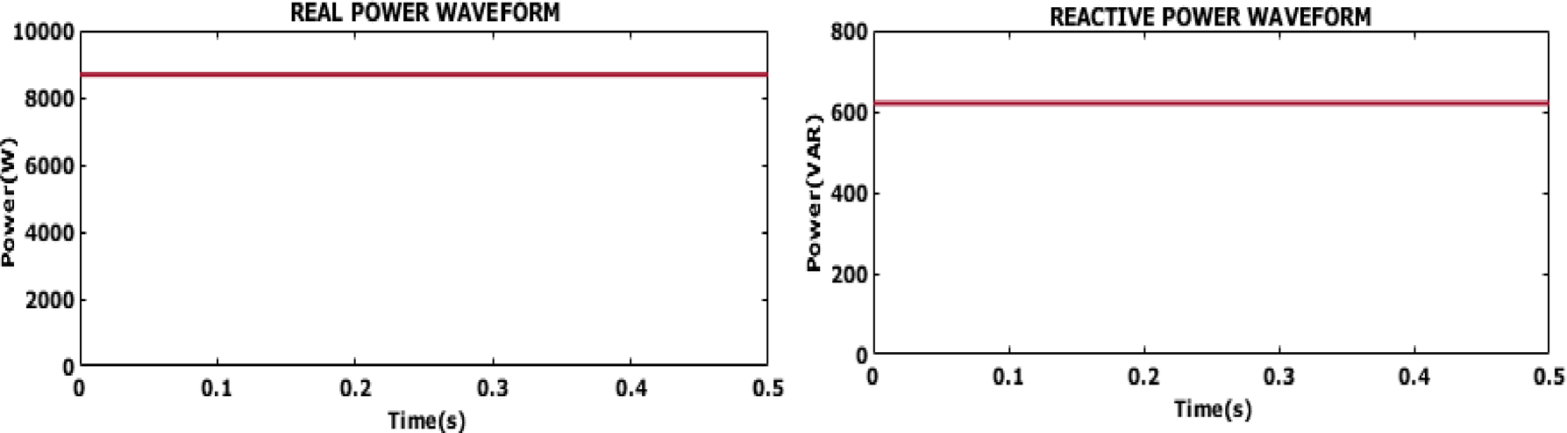
Reactive and Real power waveform.

Figure 23 displays the waveforms for reactive and actual power. The waveforms show that the system maintains a constant real power output of approximately 8500 W, indicating efficient and steady power delivery. The reactive power remains low at around 625 VAR, suggesting minimal non-useful power generation and a high-power factor. This balance highlights the system’s effectiveness in optimizing power generation and ensuring efficient operation.

**Fig. 24:**
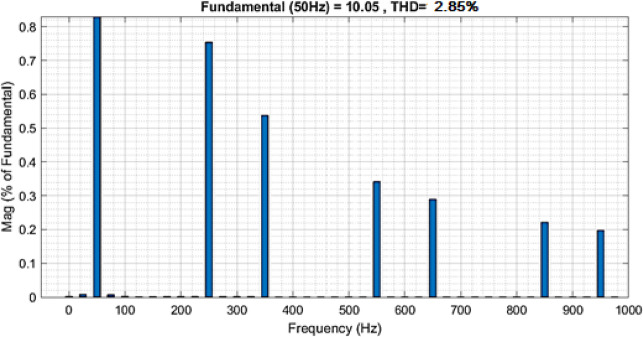
Grid current THD waveform.

The frequency spectrum waveform in Fig. 24 shows the harmonic content of the system’s output signal, with the fundamental frequency set at 50 Hz. The Total Harmonic Distortion (THD) is calculated to be 2.85%, which is relatively low, suggesting that the system maintains a high-quality output with minimal distortion. A THD value below 5% is generally considered acceptable in power systems, indicating that the waveform is close to a pure sinusoidal form. The low THD in this spectrum demonstrates the effectiveness of the control strategy in minimizing harmonic distortion, which is crucial for reducing power losses, preventing overheating in electrical components, and ensuring efficient and reliable operation of the power system.

**Fig. 25:**
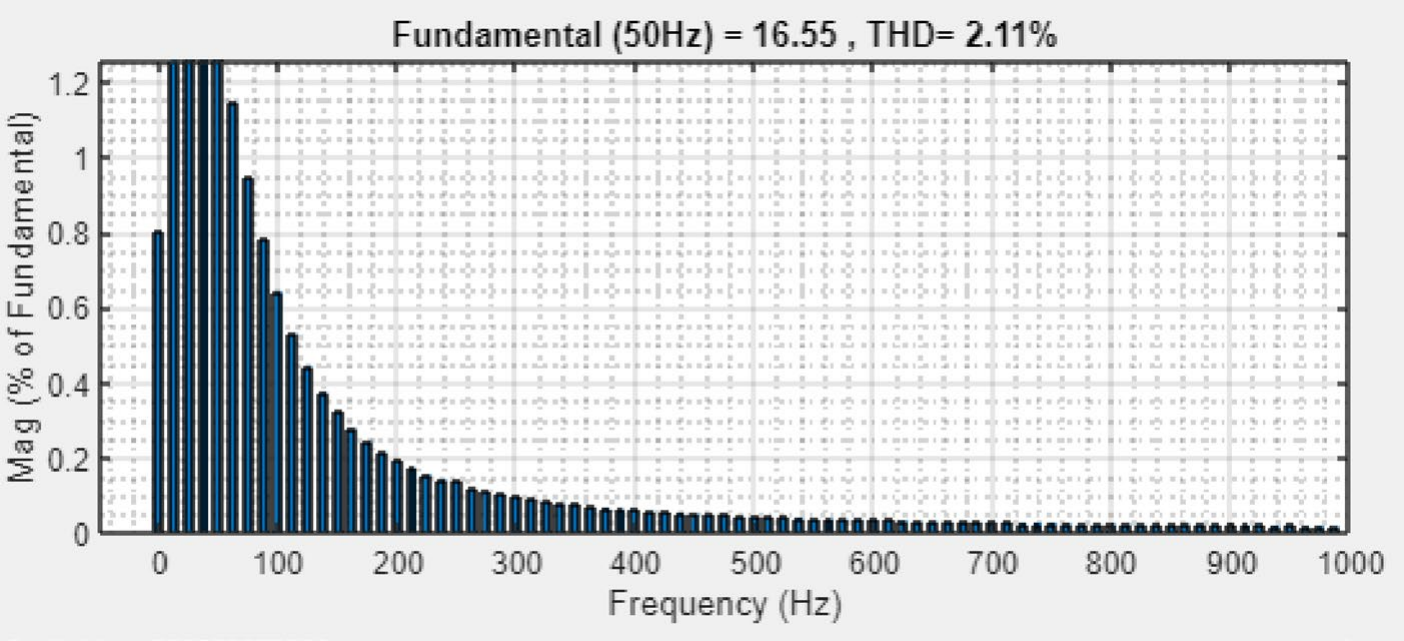
AC voltage THD waveform.

The AC voltage THD waveform is illustrated in Fig. 25, which has the lowest THD value of 2.11%, thereby enhancing the overall performance of system, improving power quality leads to efficient energy conversion than conventional approaches.

**Table 3:** Comparison of tracking speed.

Control Techniques	Tracking speed in time (S)
M-PSO^27^	3.4
I-GWO^28^	0.24
ANFIS-MPPT^29^	0.21
CSSO-ANFIS MPPT	0.08

**Fig. 26:**
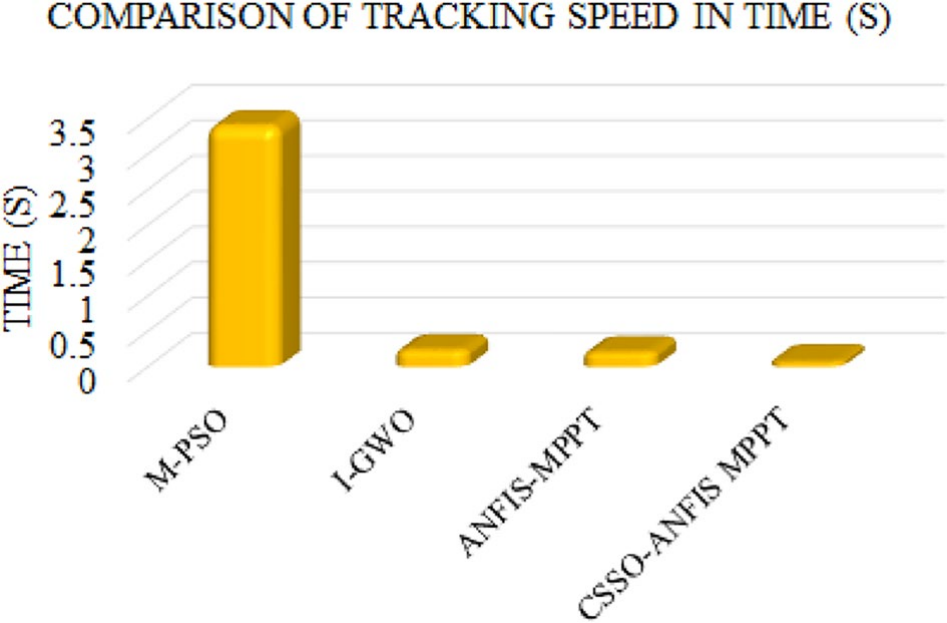
Comparison of tracking speed in time (s).

The chart in Fig. 26 compares the tracking speeds of various MPPT methods in seconds. The M-PSO method has the longest tracking time to reach the MPP, which indicates slower convergence. In contrast, the I-GWO, ANFIS-MPPT, and CSSO-ANFIS MPPT methods demonstrate significantly faster tracking speeds, all under 0.5 s. Among these, the CSSO-ANFIS MPPT shows the quickest tracking speed, demonstrating its capability to rapidly and efficiently adjust to conditions and reach MPP. This comparison highlights the superior performance of the CSSO-ANFIS MPPT in achieving faster tracking, which is crucial for optimizing energy capture in dynamic wind conditions.

**Fig. 27:**
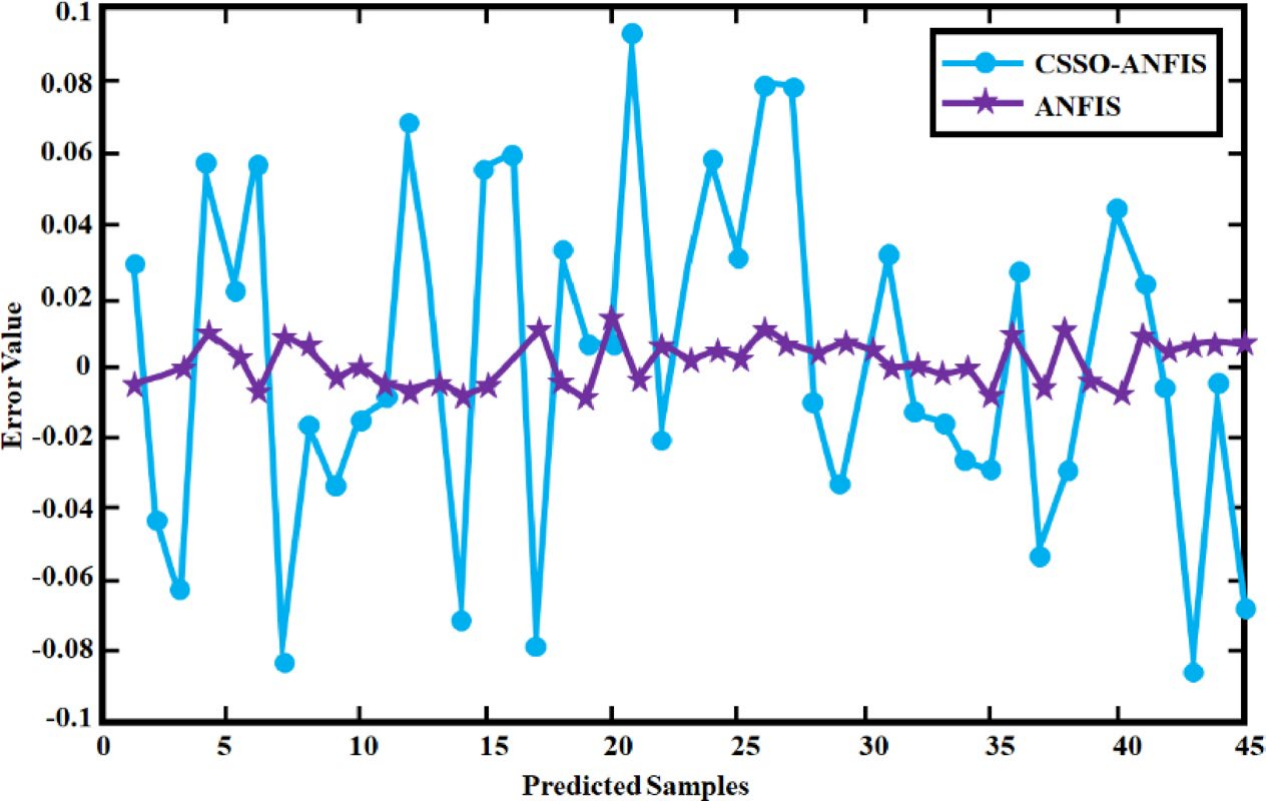
comparison of Error value.

The Fig. 27 demonstrates that the CSSO-ANFIS model exhibits a notably reduced prediction error relative to the standalone ANFIS^28^ model across the range of samples. The error values for the CSSO-ANFIS model are consistently closer to zero and display less fluctuation, implying enhanced prediction stability and accuracy. The reduced variability in error suggests that the integration of CSSO with ANFIS enhances the model’s robustness against the variability inherent in wind energy data. The overall pattern indicates a clear improvement in performance when employing the CSSO algorithm in conjunction with ANFIS for predictive tasks within the context of WECS.

**Fig. 28:**
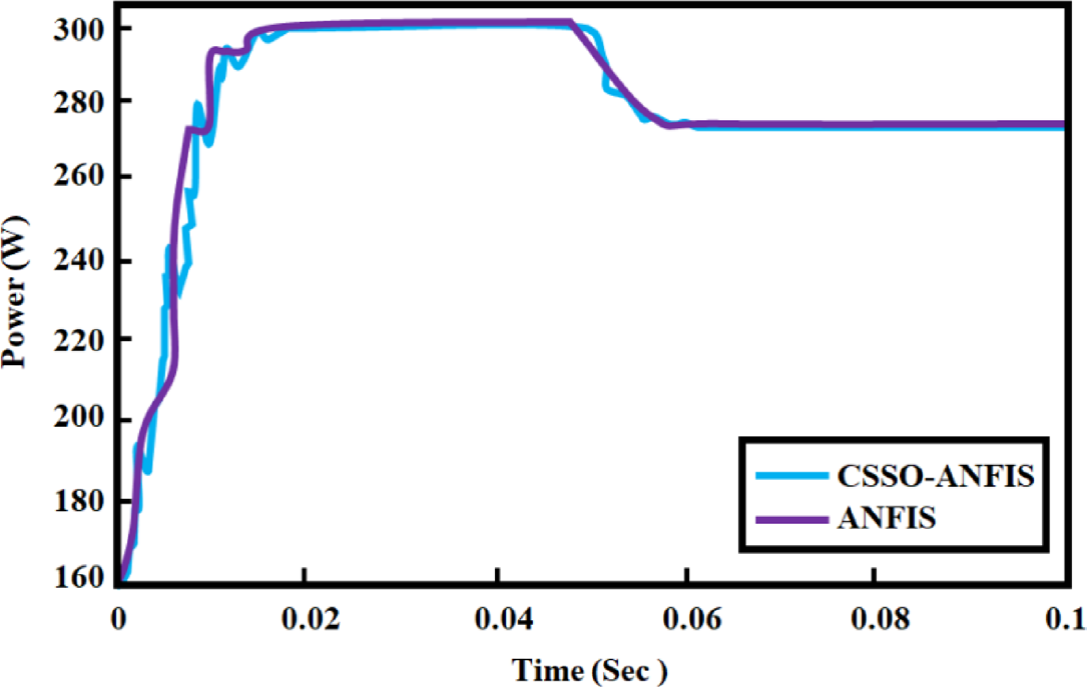
Power enabling MPPT.

The Fig. 28 illustrates the power output over time for two Maximum Power Point Tracking (MPPT) techniques: one utilizing ANFIS^25^ and the other using CSSO-ANFIS. Both techniques exhibit a rapid increase in power output as time progresses from 0 to approximately 0.02 s, where they reach a similar maximum power value near 300 W. After this point, the power output for both techniques, indicate a stable operation at the MPP. The CSSO-ANFIS MPPT curve shows less fluctuation around the MPP compared to the ANFIS MPPT curve, which implies that CSSO-ANFIS offers more stable power output in this scenario.

**Fig. 29:**
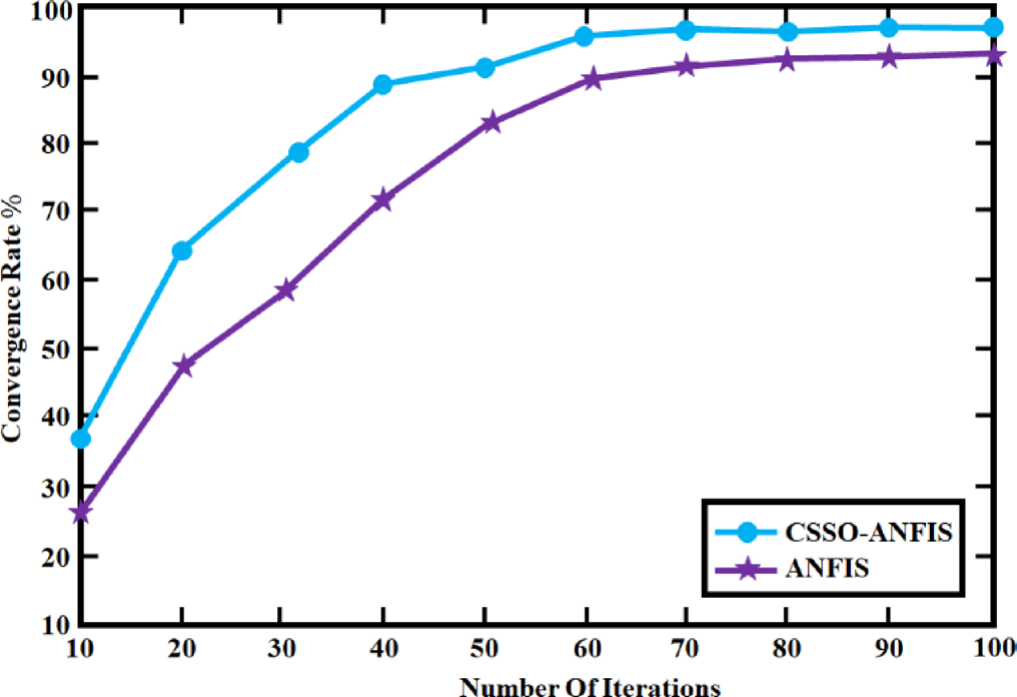
Comparison of convergence rate.

The rates at which the two control strategies, CSSO-ANFIS and ANFIS^25^, converge over a range of iterations are shown in Fig. 29. Approximately 50 iterations are needed for CSSO-ANFIS to reach 100% convergence, and 60 iterations are needed for ANFIS. This proposes that, under these conditions, ANFIS converges more slowly than CSSO-ANFIS.

**Table 4:** Evaluation of MPPT in wind energy systems.

Ref	MPPT	Year	Efficiency	Tracking speed	Oscillations
^70^	Lookup table	2018	95	0.01	0.02
^71^	CFPA	2019	99.8	1.2	negligible
^72^	P&O- Lookup table	2020	98	0.005	Less than 2 W
^61^	SSA	2020	99.3	0.2	Less than 1 W
^62^	MGWO	2021	99.35	1	Low
Proposed	CSSO-ANFIS	-	99.86	0.08	negligible

The Table 4 presents various Maximum Power Point Tracking (MPPT) methods along with their respective efficiencies, tracking speeds, and oscillation levels. In^33^, a lookup table achieved an efficiency of 95% with a tracking speed of 0.01 and oscillations of 0.02. The CFPA method^29^, demonstrates a significantly higher efficiency of 99.8% with a tracking speed of 1.2 and negligible oscillations. In^30^, a P&O-lookup table combination achieved an efficiency of 98%, a tracking speed of 0.005, and oscillations less than 2 W. Another method SSA^31^, achieved an efficiency of 99.3% with a tracking speed of 0.2 and oscillations less than 1 W. The MGWO method^34^ achieved an efficiency of 99.35% with a tracking speed of 1 and low oscillations. Finally, a proposed method using CSSO-ANFIS achieved an efficiency of 99.86%, a tracking speed of 0.08, and negligible oscillations.

The comparative analysis of MPPT techniques implemented in MATLAB reveals that the CSSO-ANFIS method outperforms other methods across every evaluated metrics. It achieves the highest power capture efficiency of 99.86%, the fastest tracking speed at 0.08 s, and the lowest THD of 2.85%, indicating superior power quality. Additionally, it exhibits the quickest transient response of 0.1 s and ensures excellent grid stability. Advanced methods like MPC, ANN, and ANFIS also demonstrate high performance, particularly in terms of efficiency and stability, making them viable alternatives. In contrast, traditional techniques such as the PI Controller and P&O exhibit lower efficiency, slower tracking speeds, and higher THD, underscoring the advantages of utilizing advanced MPPT strategies like CSSO-ANFIS in modern wind energy systems for enhanced performance and reliability.

**Table 5:** Comparison of sensitivity analysis.

Population size	CSSO-ANFIS			SSO-ANFIS		
	RMSE	*R* ^2^	VA	RMSE	*R* ^2^	VA
10	0.0556	0.8321	0.7954	0.0652	0.7824	0.7511
20	0.0531	0.8497	0.8132	0.0628	0.7935	0.7638
30	0.0512	0.8641	0.8256	0.0614	0.8012	0.7745
40	0.0497	0.8703	0.8378	0.0614	0.8005	0.7745
50	0.0485	0.8758	0.8423	0.0609	0.8051	0.7798

The performance metrics considered for the sensitivity analysis shown in Table 5 are the root mean square error (RMSE), maximize reliability (R^2^) and variance account (VA). Here, RMSE evaluates the accuracy of the model predictions, R^2^ denotes the reliability of the model predictions and VA measures the variance account providing a measure of the model’s predictive robustness. The CSSO-ANFIS exhibits a consistent performance by demonstrating lower RMSE values compared to SSO-ANFIS indicating better accuracy in prediction. The R^2^ values of CSSO-ANFIS are higher which in turn reflects a stronger correlation between predicted and actual outputs. Moreover, it also attains better VA outcomes denoting superior variance explanation in data.

**Fig. 30:**
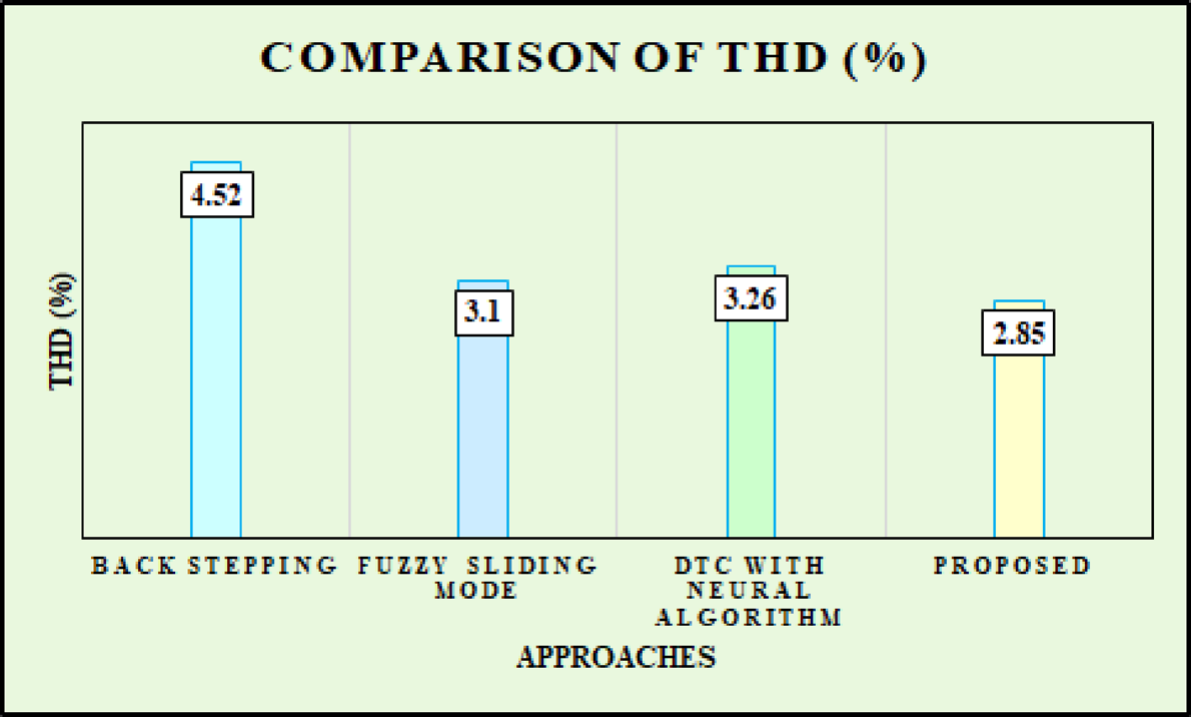
Comparison of THD.

Figure 30 represents the comparison of THD for back stepping^23^, Fuzzy sliding mode^35^, Direct Torque Control (DTC) with neural algorithm^36^ and developed approach. The back Stepping approach has the maximum THD at $\:4.52\text{\%},$ representing inferior harmonic performance. Fuzzy sliding mode and DTC with neural algorithm methods has better results, with THD values of $\:3.1\text{\%}\:and\:3.26\text{\%}$ respectively. The developed approach attains the lowest THD at $\:2.85\text{\%},$ replicating its superior ability in diminishing harmonic distortion. In the developed CSSO based ANFIS-MPPT for DFIG, sensitivity analysis is employed to assess adaptability and robustness of the system under changing environmental or input conditions. It emphases on variations in air density, rotor speed, wind speed and system parameters impact the key performance outcomes like voltage stability, power output, harmonic distortion and tracking efficiency. The chaotic strategy of the CSSO algorithm is tested for its capability to evade local minima and quickly adapt to variations. The system’s response validated exceptional resilience, with the CSSO-ANFIS MPPT controller attaining a steady converter output voltage within 0.1 s in dynamic wind conditions than slower responses from conventional methods. Moreover, the developed system sustained low power output oscillations and minimized harmonic distortion, as proved by a grid-side current THD of $\:2.85\text{\%},$ well within acceptable limits. These results confirm that the developed controller offers greater adaptability and robustness to environmental and operational disturbances, assuring both maximum energy extraction and steady grid incorporation even under rapidly changing conditions. Finally, the sensitivity analysis highlights the efficacy of incorporating chaotic optimization with adaptive neuro-fuzzy inference to address the inherent randomness of wind energy systems.

### Challenges in real-world implementation

The hardware constraints including the need for high-speed processors and sufficient memory creates impacts in real-time processing. Moreover, system reliability under extreme environmental conditions, grid disturbances and voltage fluctuations leads to risks in stable operation creating control failures. Added to this, potential failure modes like communication delays, sensor inaccuracies and converter malfunctions affect grid compliance and power quality. These issues can be mitigated by robust hardware selection and thorough testing under diverse conditions for ensuring reliable and safe deployment in wind systems.

### Conclusion

This research presents an intelligent and robust control strategy for DFIG-based Wind Energy Conversion Systems (WECS), combining a Chaotic Salp Swarm Optimization (CSSO) algorithm with an Adaptive Neuro-Fuzzy Inference System (ANFIS) for Maximum Power Point Tracking (MPPT), and a coordinated control framework for Rotor Side Converter (RSC) and Grid Side Converter (GSC). The proposed system was rigorously validated using MATLAB/Simulink under both constant and varying wind conditions. The CSSO-ANFIS MPPT demonstrated superior tracking performance with a tracking speed of 0.08 s—outperforming benchmark methods such as M-PSO (3.4s), I-GWO (0.24s), and traditional ANFIS-MPPT (0.21s). Additionally, the proposed approach achieved high power extraction efficiency of 99.86% and exhibited negligible oscillations around the maximum power point. The coordinated control strategy effectively regulated the DC-link voltage and managed active and reactive power flows, while maintaining grid voltage and current in-phase, thereby improving the power factor to near unity. Harmonic mitigation was successfully achieved, with a Total Harmonic Distortion (THD) of 2.11% in AC voltage and 2.85% in grid current—both well below the IEEE-519 limits. Comparative THD analysis with methods such as backstepping, fuzzy sliding mode, and DTC with neural control highlighted the proposed system’s superior performance in power quality enhancement. Furthermore, sensitivity analysis under varying wind speeds and environmental conditions confirmed the controller’s adaptability and robustness, ensuring stable operation and optimal energy capture even under dynamic scenarios. These findings confirm that the proposed CSSO-ANFIS and RSC-GSC coordinated control framework not only enhances the efficiency and reliability of wind energy systems but also supports grid stability and power quality.

## Data Availability

No datasets were generated or analyzed during the current study.
